# A Century of Change in Kenya's Mammal Communities: Increased Richness and Decreased Uniqueness in Six Protected Areas

**DOI:** 10.1371/journal.pone.0093092

**Published:** 2014-04-09

**Authors:** Anikó B. Tóth, S. Kathleen Lyons, Anna K. Behrensmeyer

**Affiliations:** Evolution of Terrestrial Ecosystems Program. Department of Paleobiology, National Museum of Natural History, Smithsonian Institution Washington, District of Columbia, United States of America; Institute of Agronomy, University of Lisbon, Portugal

## Abstract

The potential for large-scale biodiversity losses as a result of climate change and human impact presents major challenges for ecology and conservation science. Governments around the world have established national parks and wildlife reserves to help protect biodiversity, but there are few studies on the long-term consequences of this strategy. We use Kenya as a case study to investigate species richness and other attributes of mammal communities in 6 protected areas over the past century. Museum records from African expeditions that comprehensively sampled mammals from these same areas in the early 1900's provide a baseline for evaluating changes in species richness and community structure over time. We compare species lists assembled from archived specimens (1896–1950) to those of corresponding modern protected areas (1950–2013). Species richness in Kenya was stable or increased at 5 out of 6 sites from historical to modern times. Beta-diversity, in contrast, decreased across all sites. Potential biases such as variable historical vs. modern collection effort and detection of small-bodied, rare, and low-visibility species do not account for the observed results. We attribute the pattern of decreased beta diversity primarily to increased site occupancy by common species across all body size classes. Despite a decrease in land area available to wildlife, our data do not show the extinctions predicted by species-area relationships. Moreover, the results indicate that species-area curves based solely on protected areas could underestimate diversity because they do not account for mammal species whose ranges extend beyond protected area boundaries. We conclude that the 6 protected areas have been effective in preserving species richness in spite of continuing conversion of wild grasslands to cropland, but the overall decrease in beta diversity indicates a decline in the uniqueness of mammal communities that historically characterized Kenya's varied landscape.

## Introduction

Wildlife reserves and national parks have been established around the world to protect biodiversity from environmental change and human impact, but there has been little systematic research examining the relationship between protected areas, species diversity and community structure over ecologically long time periods. The well-documented mammal fauna of Kenya provides a case study for examining biodiversity trends and assessing the impact of national parks and reserves on these trends. Many of the target ecosystems have been studied individually [1]–[10] but not in a regional framework over time. Prior studies of species diversity usually focus on a particular body size range or taxonomic group and do not provide comprehensive records of the entire mammal community.

**Figure 1:**
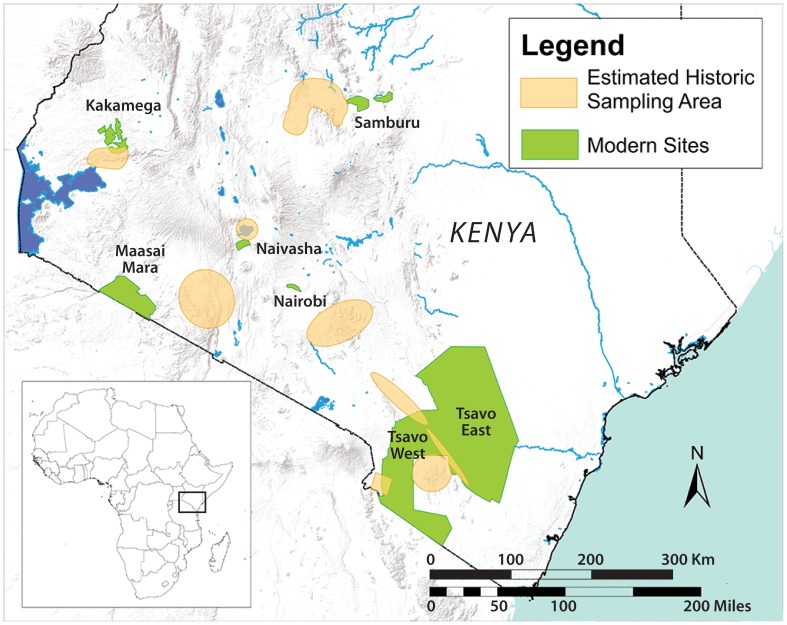
Study area. Map of modern sites with estimated areas of historical sampling superimposed (see Appendix C 2.1 in File S2 for more information). GIS data from WRI [41].

Establishing biodiversity trends requires baseline information on species present in particular geographic areas at a known time in the past. African expeditions of the early 20^th^ century included scientists and hunters who collected comprehensive samples of wildlife species from different ecosystems in Kenya. Such collections and associated documentation for both large and small mammals now represent a valuable archive of information about biodiversity and community structure in an earlier stage of human impact on wildlife. We evaluate changes in the mammal communities over the past century by comparing modern species lists from protected areas in Kenya with historical museum collection records from the same areas in the first half of the 20^th^ century. We ask how mammal species richness and other metrics of community structure (e.g., beta diversity, body size distributions, trophic structure) have fared in 6 protected areas (Fig. 1). These wildlife reserves and parks were established in the mid 1900's, and comparing mammal communities before and after 1950 provides a test of the effects of increasing human activity and environmental change [2], [7]–[9] on these ecosystems.

**Figure 2:**
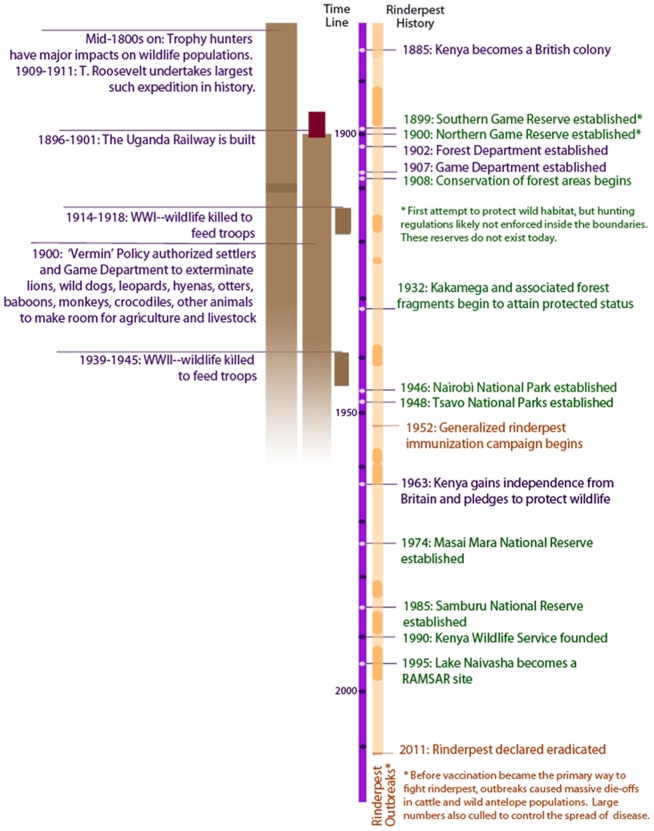
Kenya's wildlife policies. A timeline summarizing Kenya's policy on natural resources and wildlife over the past 120 years [11], [18].

The history of Kenya's wildlife policies provides a useful background for understanding various anthropogenic pressures on the country's mammal communities, particularly those of our 6 study sites (Fig. 2). In the 1890's, the British government adopted a policy to protect the colony's natural resources and also built the railroad from Mombasa to Uganda, which brought large numbers of white settlers and hunters into the Kenyan highlands. A Forest Department was established in 1902 and a Game Department in 1907. In the following decades, most of Kenya's major forests came under government protection. Kakamega Forest and its associated fragments were designated in the 1930's, the earliest protected status for any of our 6 study sites. The Game Department simultaneously put into place a “vermin” policy, allowing extermination of animals such as lions, leopards, hyenas, wild dogs, otters, baboons, monkeys, and crocodiles, both inside and outside newly formed protected areas. This policy, in conjunction with expeditions by white big-game hunters and increasing settler-wildlife conflicts, resulted in widespread loss of wild animals in the first half of the 20^th^ century [11]. By the 1930's and 40's, the British government recognized the need for more effective wildlife protection. Local campaigns to protect wildlife progressed through the 1930's but were delayed by WWII, when large numbers of wildlife were hunted to feed troops in Africa [11]. Nairobi National Park was established in 1946, followed by Tsavo East and West National Parks in 1948. The Maasai Mara, historically used for ranching by local people, was designated as a Reserve in 1974, Samburu not until 1985. Lake Naivasha was not designated a protected area until it was recognized as a Ramsar site and a wetland of international importance in 1995 [12]. Today, all of these areas are under the jurisdiction of the Kenya Wildlife Service, which oversees wildlife monitoring, conservation, tourism, anti-poaching and wildlife-human conflict resolution in and around protected areas.

We use this system of protected areas in Kenya, along with associated information on species richness, size of the protected areas, human population and cropland density across the last 100 years, as a case study to investigate changes in mammalian community structure over time. Specifically, we evaluate changes in species richness, beta diversity, body size and trophic distributions. We compare our results to possible causal factors including sampling issues, changes in dominant habitat type, and the movement of species into and out of protected areas. Finally, we discuss our results in light of predictions from species-area relationships and the role of anthropogenic effects.

## Materials and Methods

We selected sites in Kenya using the following criteria: 1) historically surveyed areas corresponding as closely as possible to modern-day national parks or reserves (Fig. 1, see Appendix C in File S2 for more information on the comparability of modern and historic collecting areas), and 2) historic records from the Smithsonian African expeditions contained at least 200 individual specimens for each site (see Table S1 in File S1). Historical and recent place names were reconciled using archival maps and Google Earth. Our sites are located throughout central, western, and southern Kenya and comprise a diverse range of habitat types, including forest (Kakamega Forest Reserve), grassland (Maasai Mara National Reserve), savanna (Nairobi National Park, Samburu Game Reserve), wetland (Lake Naivasha National Park), and woodland/scrub habitat (Tsavo East and West National Parks) (Table 1). For each site, we compiled non-volant, non-domestic mammal species lists from two time periods: 1896–1950 and 1951-present. We obtained mammal specimen and sighting data from a variety of sources, including specimen-based records from the Smithsonian African Expedition and 13 other museums through the Mammal Networked Information System [13], [14], the Global Biodiversity Information Facility (GBIF) [15], photographs, published literature, and databases [16]–[32] (see Appendix A in File S2). Taxonomy for all records was updated and standardized according to the latest version of Wilson and Reeder's mammalian taxonomy [25]. Differences in the original taxonomic identifications of species compared with present classification affected about 10% of the final species names listed, and most were easily and consistently resolved (Appendix B in File S2).

**Table 1:** Site information and gamma-diversity counts.

Site	Year protect.	Modern Area (km^2^)	Estimated Hist. Area (km^2^)	Mod. species	Hist. species	Shared species	Main Habitat Type	Main Stressors
Kenya	—	582,650	582,650	208	192	156	—	—
Kaka-mega	1936	540	800	87	61	36	Forest	Very heavy pressure from logging, population growth, agriculture on west side
Maasai Mara	1974	1510	1300	107	74	58	Grassland, Savanna	Heavy pressure from ranching, industrial farming in Loita Plains
Nairobi	1946	120	700	100	94	70	Savanna	Heavy pressure from city development, but fenced and well-protected
Naivasha	Not protectedRamsar 1995	200	800	76	88	49	Wetland	Very heavy pressure from floriculture, ranching, agriculture
Samburu	1985	430	2500	88	76	59	Riverine/scrub	Moderate to low pressure from ranching and population increase
Tsavo	1948	20,000	4000	123	81	55	Bush/Scrub	Moderate pressure from population increase, poaching

Modern areas calculated based on the size of modern protected areas. Historical areas were calculated from geo-referenced historical maps of the Roosevelt expedition. M = modern; H = historical; Shared H&M = number of species that occurred in both time periods; Total H+M  =  total number of species recorded in any time period without overlaps.

For each site, we also calculated the size of the modern protected area using ArcGIS 10.1 [33]. Using the same program, we georeferenced maps of the historical sampling areas and used these to estimate the corresponding historical areas sampled by early expeditions. Because our historic sites represent sections of wilderness largely unaffected by settlement (with the exception of pastoralist societies in some localities, which did not exclude wild animals), we assumed that the historical areas are a reasonable estimate of the minimum available areas for wildlife before 1950. We compared these to the areas of the corresponding modern protected areas, which represent the only remaining land areas where human impact is minimal. This comparison produced a conservative estimate of the loss of land available to wildlife between the two time periods.

We extracted information on population density and agricultural intensity from the HYDE database (History Database for the Global Environment) [34], [35] for the time period 1900 and 2000 to evaluate how land use and human impacts have changed over the last century in and around these protected areas (Fig. S1). The HYDE database estimates information on land use and population density for the last 12,000 years based on the IMAGE model [36], which simulates the effects of human activities worldwide. Using the information on population density and agricultural intensity, we estimated the change in land use in a 15 km buffer zone around each sampled site to evaluate increased pressures on wildlife populations outside these areas over the last century.

### Size and Visibility Classes

Charismatic and large-bodied mammals tend to be easier to observe and identify and often receive more scientific attention than other species. We address this potential bias in the species richness data by comparing data subsets for which we have varying levels of confidence. We divided all the species data into three size classes: Small  = 0–5 kg, Medium = 5–10 kg, Large >10 kg. If the resulting patterns of species occurrence are similar across these three size classes, this will indicate that the signal in the data is not being driven by better sampling of large-bodied species than small-bodied species. Even if equal efforts were directed toward collection of all size classes, the visibility or “detectability” of species not typically recorded using standard trapping methods could still introduce sampling bias against, solitary, cryptic, low density, or nocturnal species. We assigned species in the large- and medium-bodied categories a visibility rating (high, medium, or low) using a qualitative assessment of species behavior, habitat, social structure, and body size. For example, common, conspicuous savanna species such as *Aepyceros melampus* (impala) and *Panthera leo* (lion) were classified as high visibility, while nocturnal and solitary large species as well as common smaller species, such as *Canis mesomelas* (black-backed jackal) and *Procavia capensis* (rock hyrax), were classified as medium. Low visibility species included small carnivores, nocturnal medium-bodied species, and dwarf antelopes such as *Cephalophus* (duiker). We excluded small mammals from this analysis because the probability of collecting them in traps does not depend on visibility, but rather population density and other factors. The large-bodied, high visibility category represents the subset of species for which we have the greatest confidence regarding presence or absence in both the historical and modern data. Similarities in the resulting patterns of occurrence across visibility classes will indicate that our results are robust with respect to potential sampling problems, whereas differences will indicate that these results may be driven by changes in a specific subset of the species pool, or by biases in sampling.

### Richness and Beta Diversity

We evaluated the change in species richness across time for each site using a paired t-test. We also calculated beta diversity using the Sorensen index for all possible pairs of sites to compare across space within each time period. This index measures similarity (the inverse of beta-diversity) between 2 sites using a ratio of overlapping species and the total species counts. We also calculated the index for historical and modern species lists from the same sites to measure the change in beta diversity across time. We used the Wilcoxon Signed-Rank test and paired t-tests to determine whether beta diversity in historic Kenya was significantly different from that of modern Kenya. We ran another set of analyses with Kakamega excluded from the data because that site experienced the strongest faunal changes, and it was important to verify that turnover at a single site was not the main driver of the observed changes. We repeated these analyses using our size and visibility classes to test whether patterns differed among different subsets of the data, and whether any of these patterns suggested a sampling bias.

### Body Size, Trophic Distributions, and Vegetation Patterns

Ecological changes such as distributions of functional traits provide information concerning the general structure of communities over time. To evaluate changes in the ecological function of the target communities, we used information on body size and trophic level (diet) for all species taken from an updated version of Smith *et al*. [37]. We compared body size and trophic distributions of each site over time and across space within each time period using Kolmorgorov-Smirnov two sample tests. A substantial body of research on cenogram analyses indicates that the shape of the body size distribution is also correlated with the dominant habitat type [38]–[40]. Thus a significant shift in the body size distribution can indicate shifts in habitat, (e.g., an increase in proportion of large mammals corresponds to more open habitats). Drawing on this research, we examined the body size distributions for indications of shift in vegetation. When any such shift was indicated, we searched archives and the literature for independent evidence of a directional vegetation shift across our time periods that could help explain this body size change [4], [26]–[30], [41]. We used this methodology because we were unable to find adequate quantitative information on vegetation within the sites for a direct comparison between the two time periods.

### Species Movements

To evaluate the observed changes in terms of the species identities, we calculated which species were driving the changes in richness and beta diversity. We counted species showing each possible change in occupancy (total number of occupied sites, Table S2 in File S1) and weighted each category with the net change in species overlap for the 15 possible pairs of sites. For example, if one species starts in 2 sites and colonizes 1 additional site, this increases similarity between the 2 initial sites and the new site (two pairs), but decreases similarity between the new site and those where the species remained absent (three pairs). The remaining pairs are unaffected, and the net effect of the occupancy change is -1. Seven species exhibited this behavior, so the net impact is -7 (Table S2 in File S1). The net impact values can be plotted on a three-dimensional surface, where peaks show the largest net impact.

### Collection curves

Following precise collection dates recorded by J. Loring, E. Mearns, and E. Heller [26]–[30], we constructed collection curves for historic specimens acquired by the Field Museum East Africa Expedition (1905–1906) and the Smithsonian African Expedition (1909–1911). These expeditions spent intervals of weeks to a few months at a site and often returned after longer intervals, in part so that they could sample during different seasons (e.g., dry vs. wet). When specimen collection at a site ceased for ten days or more between collections, we considered this interval outside of the sampling effort and removed the duration between the two collections that occurred during this time. If historic sampling was thorough, the collection curves should level off by the end of the collecting period, as fewer and fewer new species were found.

### Population Density vs. Occupancy

As an additional strategy to detect sampling biases, we used the presence/absence data in our 6 sampling areas to calculate historical and modern occupancy (percent of occupied sites) for each species. Using population density values available for 122 of our 244 species [42], we plotted change in occupancy from the historical time period to the modern against population density as an independent estimate of rarity, calculating separate regressions for large, medium, and small mammals. If historical sampling was inadequate, we would expect historic surveys to miss rare species but record the more common ones. If these rare species were and are present in a site, and were better sampled by recent censuses, then they would show up in the modern records, thereby inflating the sampled occupancy over time. As a result, we would expect to see a systematic bias of increasing occupancy in rarer mammal species, while widespread and common species remain relatively stable. If sampling in modern surveys was inadequate, we would expect the opposite pattern, with rare species decreasing occupancy, while common species remained relatively stable.

## Results

We collected data from 6 sites totaling an estimated 10,000 km^2^ area in the historic and 23,000 km^2^area in the modern record (Table 1). The difference in area is due mainly to the size of the modern Tsavo National Parks (East and West). The remaining 5 sites show an estimated average decrease of 69% in available protected land area for wildlife between the historical areas sampled and the modern protected areas. The current Tsavo National Parks cover 20,000 km^2^, although the historical and modern species lists likely represent sampling of comparable areas (about one-fifth of the current park size). At the same time that land area available for wildlife was decreasing, the human population density in Kenya and the amount of land devoted to cropland was increasing (Fig. S1). The change in population density and cropland density in the 15 km buffer around each park demonstrates the increasing pressure from anthropogenic effects over the last century (Fig. S2).

In the historic lists, 192 species were recorded across all sites (gamma-diversity), and 208 were recorded in the modern lists, for a total of 244 species. Of these species, 156 were shared between time periods, while 52 species appeared and 36 species disappeared. Although the vast majority were small bodied, these species were not characterized by particular ecological traits and were not significantly different from the overall distribution of ecological traits across the dataset as a whole. Among the larger species, a few that appeared are understudied (*Profelis aurata*), elusive (*Manis temminckii*) or introduced (*Beautragus hunteri, Ceratotherium simum*). Of the 52 species that appeared in the modern record, 10 are taxonomically uncertain, compared to 4 of the 36 species that disappeared. Of the remaining 42 species that appeared, 34 exist outside or near the edges of their ranges at our sites, or only in small, patchy distributions, compared to 29 of the remaining 32 that disappeared. Only 6 out of all species that appeared and 3 out of all species that disappeared are reasonably common and widespread in Kenya based on range alone [32].

### Richness

**Figure 3:**
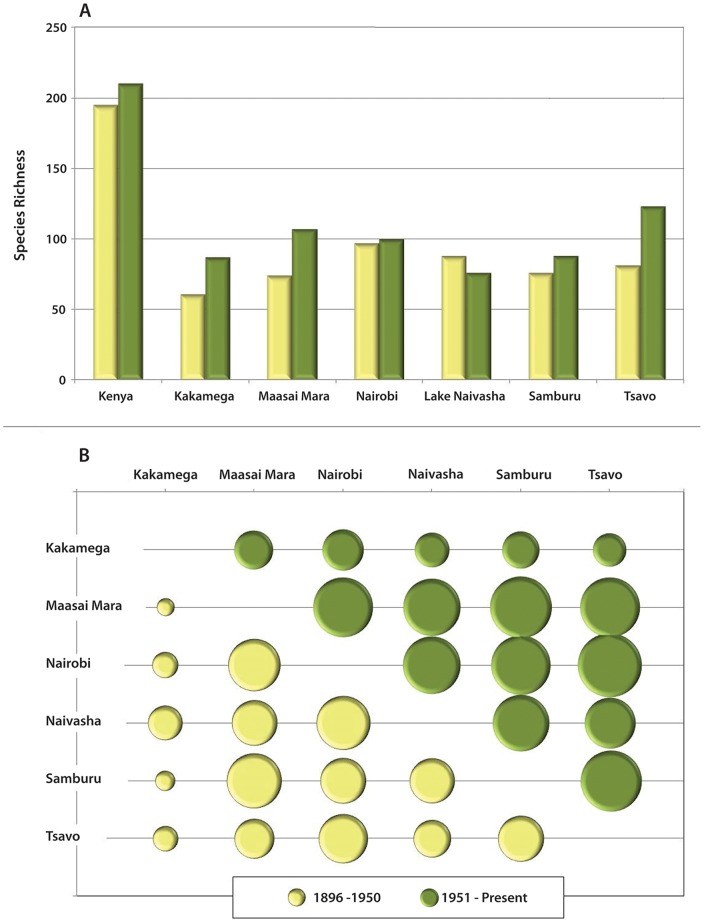
Richness and β-Diversity. (A) Comparison of species richness by site. Color code: yellow = 1896–1950, green = 1950–2013. A paired t-test indicates this is a significant increase in richness when considering all sites (t = 2.215, p = 0.039). (B) Degree of similarity between each pair of sites in the historical and modern records, using the Sorensen Index. Size of filled circles indicates degree of similarity for each pair (See Table S3 in File S1 for exact values). Comparisons of circles in different time periods show an increase in similarity for each site pair, thus a decrease in beta-diversity (Table S4 in File S1). Wilcoxon signed-rank test: p<0.0001.

We detected a net increase in species richness at 5 out of 6 sites (t = 2.215, p = 0.039) (Fig. 3A). Changes were spread over species in all size classes, with Kakamega, Maasai Mara, Samburu, and Tsavo showing an increase in every size class (Fig. S3). Nairobi had an increase in richness of medium and large mammals and no change in small mammals. In Naivasha, small and medium-sized mammals decreased but large mammals increased.

### Beta Diversity

Similarity increased in 14 out of 15 paired comparisons using all species (p<0.001) (Fig. 3B). The average similarity among modern sites (0.601; Table S3 in File S1) is approximately equal to the similarity across time at individual sites (0.638; Table S4 in File S1), whereas average similarity among historical sites is much lower (0.461; Table S3 in File S1). The magnitude of changes in the Tsavo large mammal comparisons may be affected by sampling issues in the historical period. The pattern of increased similarity holds when the beta diversity analysis is repeated using size and visibility classes (Fig. S4). Most of these pair-wise comparisons continued to show an increase in similarity over time (e.g., 12 out of 15 for small mammals, 14 out of 15 for high visibility), and all p-values were highly significant (small: p = 0.0103; medium: p<0.0001; large: p = 0.0006; low visibility: p<0.0001; medium visibility: p = 0.0002; high visibility: p = 0.0006), indicating that variable sampling effort among different body sizes or visibility classes of mammals does not significantly influence our results. Moreover, these results were not driven by logging increasing areas of open habitat in Kakamega, as the increase in similarity held across all analyses when that site was omitted. Over time, the three size classes showed a marked consistency in the magnitude of increase in similarity. The visibility classes did not show similar consistency in this respect because the low visibility class had a greater increase in similarity than the medium and high visibility classes (Figure S4).

**Figure 4:**
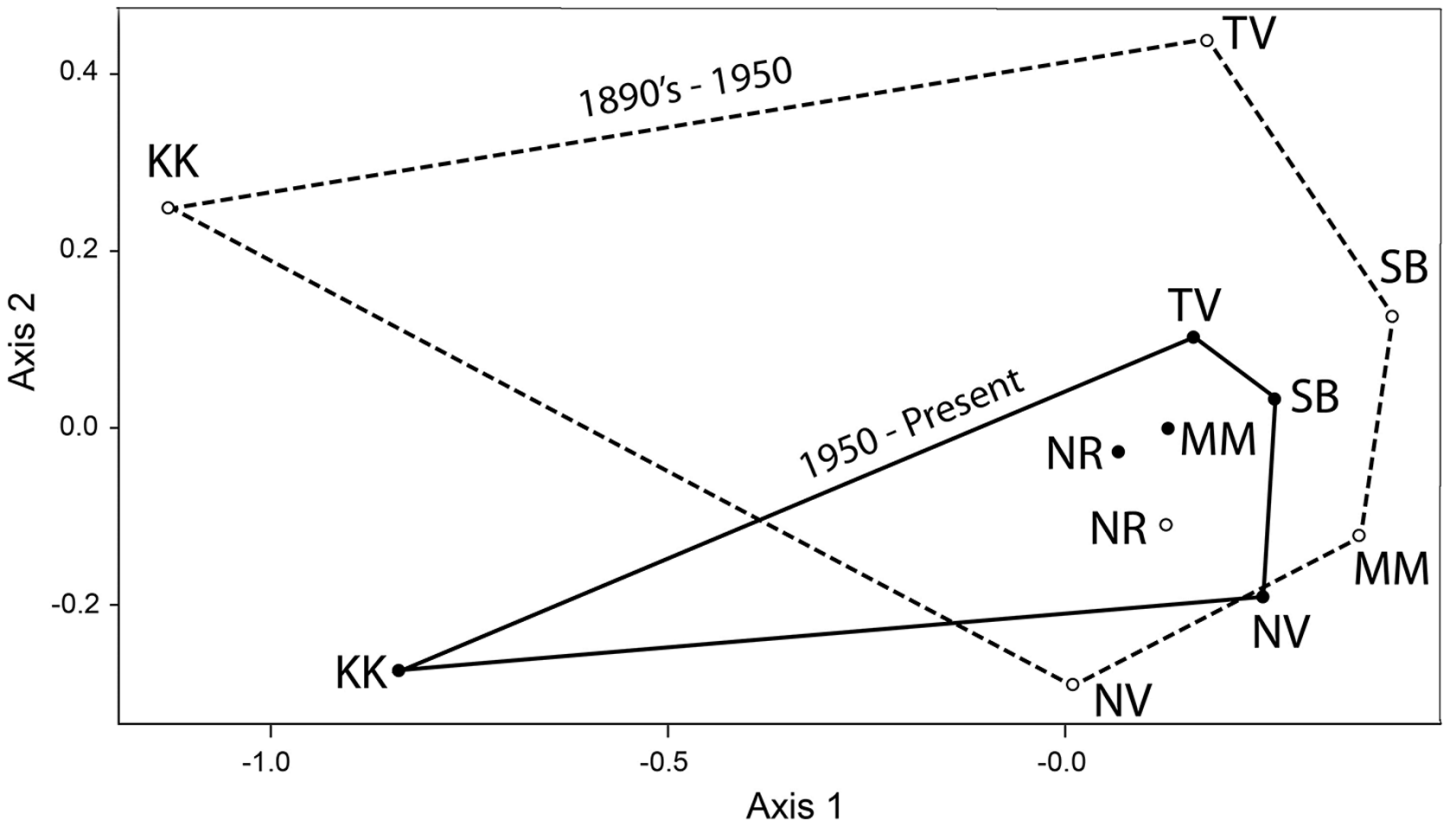
Similarity increase among sites. Non-metric multidimensional scaling (NMDS) using the Jaccard Coefficient for species lists from 6 sites (Table S3 in File S1), showing that 5 sites move closer together from historical (dashed line, open points) to the modern (solid line, filled points), and overall spread (polygons) decreases (area of historical hull = 0.629; modern hull = 0.155; Stress (av. of 4 runs) = 0.0746). Key: KK = Kakamega, MM = Maasai Mara, NV = Naivasha, NR = Nairobi, SB = Samburu, TV = Tsavo.

Nonmetric multidimensional scaling (NMDS) analysis provides a quantitative summary of the change in similarity between the historical and modern mammal species present at each site (Fig. 4). The smaller size of the polygon enclosing the modern sites indicates the increase in species the 6 sites now have in common.

### Community Composition Change

**Figure 5:**
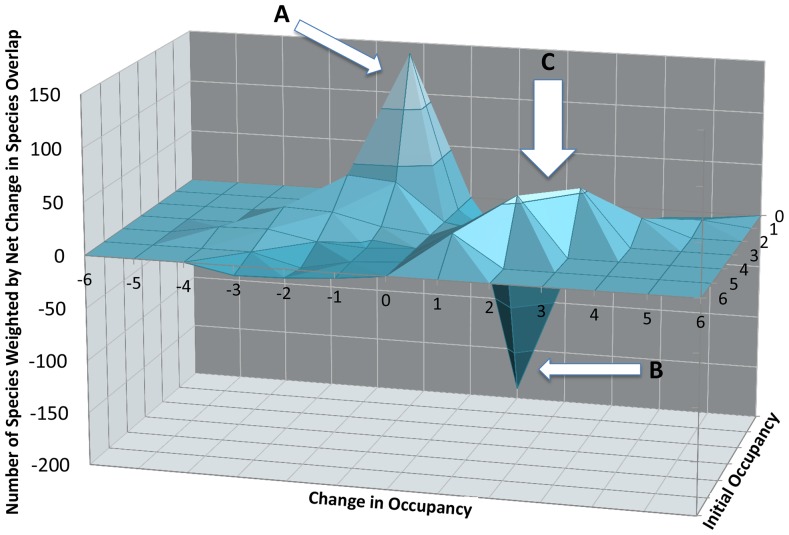
Effects of occupancy changes on β-Diversity. Net effect of occupancy change between historical and modern time intervals. The peaks represent species (A) at one site that disappeared, (B) appearing at one site and (C) originally at 2–5 sites that increased their occupancies. Peaks A and B show predicted rare mammal turnover [43]–[44], which effectively cancel each other out. Peak C includes species across all body size classes and drives the pattern of increasing similarity from historical to modern times.

Overall, 73 species decreased their occupancies, 128 species increased their occupancies, and 43 species remained the same. Since 52 species disappeared overall, a large proportion of the species that decreased also disappeared altogether. The three-dimensional surface plot (Fig. 5) shows two sharp peaks from new species appearing in only one site and existing species disappearing from their only site. These are largely the result of appearances and disappearances of small mammals and indicate a widespread local turnover of rare, satellite or transient species [43]–[44]. However, the magnitudes of gains and losses are similar and contribute little to the overall occupancy pattern, which is driven instead by another broad, positive peak caused by the occupancy increase in common species that historically occurred at 2 to 5 sites. These changes are distributed fairly evenly among large, medium, and small mammals. An analysis of the ecological trait distributions of increasing and decreasing occupancy species yielded no characteristics significantly deviating from a random sample of the data, or any marked characteristics that distinguish these two groups from one another.

### Trait Distributions and Vegetation Patterns

There were no significant differences in trophic distributions in any of the sites when compared across time periods. Only 2 sites exhibited a significant change in body size distribution, Kakamega and Naivasha (Fig. S5). The body size distribution in Kakamega shows a marked shift toward large-bodied mammals. This is consistent with evidence of increasingly open habitats due to forest clearing by humans, whose population has grown substantially to the west of the park (Fig. S1, S2 [34]–[35], [41]). In Naivasha, small-bodied mammals have decreased. While this may be partially a sampling issue, many of the missing species are at least partially dependent on forests or swamps. Some were likely visitors from the nearby escarpments in the past. This shift is also consistent with anecdotal evidence of land clearing for floriculture as well as disturbance of wetland habitats.

### Collection Curves

Visual inspection of collection curves showed that they level off toward the end of the sampling period. Some also show several rapid “jumps” within the full time period, corresponding to visits by different explorers and museums, many of which may have had different goals, collecting seasons, or collection methods (e.g., Samburu, Tsavo). For example, T. Roosevelt visited the “North Ewaso Ng'iro River” (present-day Samburu) and hunted large mammals before naturalists E. Heller and J. Loring trapped small mammals in the same area. Because the collection curves level off at all sites, this suggests a reasonably good representation of the species composition in the areas sampled during the early 20^th^ Century (Table S1 in File S1, Fig. S6).

### Population Density vs. Occupancy

**Figure 6:**
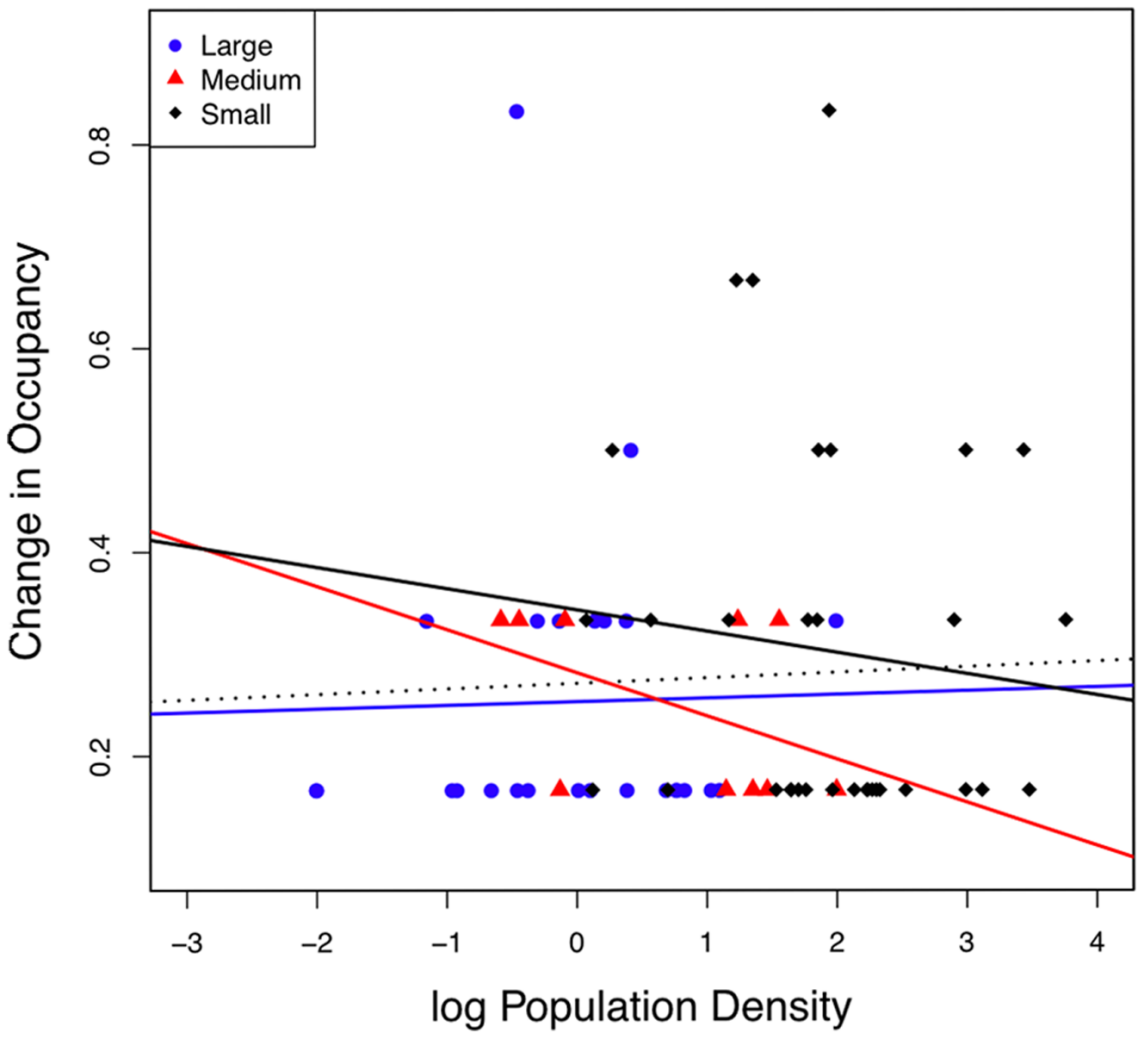
Occupancy vs. population density of pecies. Tests of the relationship of population density to increased site occupancy between the historical and modern time intervals for small, medium, and large bodied mammals. Regressions were calculated for all species (black; y = .271+.005X, R^2^ = 0.002, F = 0.125, p = .724) and small (yellow; y = .343–.021X, R^2^ = 0.011, F = 0.359, p = .553), medium (green; y = .282–.042X, R^2^ = 0.211, F = 2.144, p = .181) and large bodied (blue; y = .253+.004X, R^2^ = 4.93E–4, F = 0.011, p = .916) species separately. Data for all occupancy changes including decreases available upon request.

Poor sampling of less common species in the historical period should result in a pattern of increased occupancy post-1950 by these species (i.e., those with smaller population densities on average), and poor sampling in the modern period should result in a decrease in occupancy by less common species. Our results are not consistent with either prediction (Fig. 6, Fig. S7). Overall, there was a significant negative relationship, such that species with higher population densities were more likely to decrease their park occupancy in the modern time interval (y = 0.177–0.057X; R^2^ = 0.096, F = 12.65; p = 0.0005). However, when broken down by size class, the density-occupancy relationship was only significant for small-bodied species (small: y = 0.401–0.141X, R^2^ = 0.209, F = 16.683, p = 0.0001; medium: y = 0.271–0.055X, R^2^ = 0.205, p = 0.163; large: y = 0.117–0.017X, R^2^ = 0.005; F = 0.196, p = 0.661). This relationship was driven by more common species, (i.e., those with higher population densities), which show decreasing occupancy. This is in the opposite direction of a sampling-induced bias, which should show a greater effect from rare species. When only species with increasing occupancy are considered (the effect predicted if historical sampling was poor), there is no relationship between population density and occupancy (Fig. 6). Medium and large bodied species showed no relationship between these variables. Moreover, there were no significant relationships when we repeated this analysis with the visibility subsets (Fig. S8).

## Discussion

Our analysis shows relative stability or increase in mammalian species richness accompanied by loss in community uniqueness over all study sites across the past century. The cumulative richness of the sites did not change greatly over time, though a substantial turnover of species was observed. Our analysis showed, however, that this turnover is driven by species that have very patchy distributions or exist at the edges of their ranges in Kenya, thus are likely to have been transient at our sites. More importantly, these gamma-diversity changes are not local extirpations (other than the single noted case, *Tragelaphus eurycerus*) but merely the movement of satellite species in the system, which mainly contributes noise to the overall pattern of species richness over time.

The largest changes in beta diversity were seen in comparisons of Kakamega vs. savanna-dominant parks (Fig. 3B), presumably because historically that site supported solely forest species (i.e. medium sized arboreal and/or nocturnal rainforest mammals) but now has more open and mixed habitat species due to deforestation, whereas the remainder of the sites included open vegetation though both time intervals. The low visibility subset of the beta-diversity analysis (Fig. S4B) showed the largest difference (decrease) in beta diversity between historical and modern sites. We attribute this to the fact that some low visibility species adapted for closed, forest habitats are also ecologically specialized (e.g. *Cephalophus*), and their disappearance translates to a marked decrease in beta-diversity. Other common but low visibility taxa (e.g. *Hystrix, Mellivora, Felis silvestris*) likely have expanded by exploiting anthropogenic resource opportunities, which is similar to the pattern in other subsets. However, low-visibility species that are difficult to sample represent a small portion of the data and our analyses indicate they are not a major driver of the observed pattern. Overall, the subset analyses show that the pattern of decreasing beta diversity is robust with respect to size and visibility of species.

Although a thorough analysis of the ecological and anthropogenic pressures affecting each of our sites is beyond the scope of this study, an estimate of such pressures is provided by comparisons of the land areas available to wildlife between the two time periods (Table 1) and by examining changes in human population density and cropland density provided by the HYDE dataset (Fig. S1 and S2). If we assume that the historically sampled areas represent the minimum available for wildlife at each site, the percent decrease represents a rough quantification of the habitat loss experienced between the two time intervals. Smaller sites such as Kakamega and Naivasha have lost approximately 50% of their original area. Historically well-sampled sites such as Nairobi and Samburu have lost more than 85% of that original area. Tsavo is the only site that today protects a larger area than historical expeditions sampled. Throughout our study area, then, the stability or increase in richness is observed despite a marked loss of land areas used primarily by wildlife.

Human population density and area devoted to cropland have changed dramatically over the last 100 years (Fig. S1) [34]–[35]. This can be seen in the difference in color for the park outlines in the 1900s (Fig. S1 A & C) versus 2000 (Fig. S1 B & D). At the beginning of the historical time period, only Kakamega and Nairobi have encroaching human populations and land use (Fig. S1 A & C). However, by 2000 all the protected areas show increased encroachment from human activities (Fig. S1 B & D). Even when the protected status has kept agriculture or human populations out of the main protected area (e.g., Nairobi National Park is fenced), our analysis of a 15 km-wide band surrounding each of the protected areas shows an increase in population density and cropland in the immediate vicinity (Fig. S2).

### Possible Drivers

Given that various tests (Collection curves, Population Density analysis, etc.) indicate that sampling inconsistencies are not a significant factor driving our results, the observed patterns could be caused by: 1) vegetation change leading to more homogeneous habitats across the sites, 2) loss of rare species due to hunting or other factors, or 3) spread (range shifts) of species, including deliberate introductions or unintentional consequences of human impact.

#### Habitat Change

A directional trend toward more homogeneous vegetation across sites (e.g., from mixed forest, bush and woodland to dominant grassland, or widespread increase of irrigated agricultural land area) between our two time periods is a possible explanation for the decrease in beta diversity. Reliable quantitative data for vegetation during the historical time interval is not available at a sufficient spatial resolution or ecological detail to differentiate between our study sites or depict their temporal differences, precluding a direct comparative analysis of habitat change. Available qualitative information indicates the most change in Kakamega (deforestation/logging) and Naivasha (agriculture/floriculture encroachment, Fig. S1) [3], [32], [41], [45]. Otherwise, the remaining sites are described as grassland, woodland, and/or bush vegetation, with little evidence of major trends over the past century. Dominant habitat type influences the body size distribution of a mammal community [38]–[40], thus size distributions would be expected to change if historical vs. modern vegetation are significantly different. We used body size distributions and dietary (trophic) proxies to test for such changes in major habitat type over time, and these did not change significantly for 4 sites (Fig. S5). This indicates that any major changes in habitat and community structures: 1) were not directional, 2) did not occur across our time periods, or 3) did not occur at all. The overall consistency of the body size distributions in combination with qualitative habitat information for 4 of the sites argues against a change toward more homogenous vegetation across sites as a major driver of the observed patterns of increased richness and decreased beta diversity.

#### Loss of Rare Species

Some species that decreased occupancies, especially those that were moderately rare historically and have receded, disappeared altogether, or evaded modern sampling efforts contributed to the increased homogeneity of modern parks. These include a number of small mammals, especially *Crocidura*, but also larger species that are not tolerant of anthropogenic disturbance (e.g., *Hippotragus equinus, Hylochoerus meinertzhageni, Neotragus moschatus*). While it is true that hunting had a widespread detrimental effect on wildlife populations in the 1900s–1960s, we found no records of species that went extinct as a direct result of hunting, thus this does not influence our presence-absence data. Overall, rare species that declined or disappeared were replaced by other species with similar occupancy patterns (i.e. one occurrence), which argues against rare species as a driver of the decreasing beta-diversity pattern.

#### Spread of Common Species

The data show a net increase in site occupancy for many of the more common mammals (Fig. 5). Here we use the term ‘common’ to describe species that occur consistently in multiple parks, and this includes some that are listed as threatened, vulnerable, or endangered in the IUCN Red List [32]. Most changes at the species level involve species appearing in or leaving 1 or 2 of the 6 sites. Species are not uniformly increasing their occupancy across all sites, nor are particular sites experiencing unusual turnover. Rather, 52% of the species (128/245) are found in more sites now than historically, compared with 31% found in fewer sites (Table S2 in File S1).

The species driving the pattern of increased site occupancy between historical and modern times include large, endangered mammals such as Grevy's zebra (*Equus grevyi*), white rhino (*Ceratotherium simum*) and hirola (*Beatragus hunteri*) that have been reintroduced to protected areas. Others are mobile large mammals that could have been absent from a sampling area during the periods of historical collection because they were only occasional visitors. These include threatened species (*Lycaon pictus, Acinonyx jubatus, Hyaena hyaena, Panthera pardus, Loxodonta africana*) [46] and common species with declining population trends (*Crocuta crocuta*, [32]). Shifts in other species ranges suggest responses to various types of environmental disturbance, such as droughts (*Oryx beisa*, [47]) and deforestation in Kakamega (*Colobus guereza*, *Syncerus caffer*, *Papio anubis, Sylvicapra grimmia*, all of which inhabit disturbed forest and forest mosaics [32], [48]–[49]). Species in a number of genera have likely found new resource opportunities in human-inhabited areas that include garbage dumps, crops, and livestock, (e.g., *Civettictis civetta*, *Mellivora capensis*, *Chlorocebus, Hystrix*, *Muridae*, *Papio*, and others [32], [50]–[51]). Some genets and mongooses (*Herpestes, Genetta, Ichneumia, Atilax, Mungos*) also have adapted to human habitation [23]. All of these species were found in 2–5 of our sites historically, proving that the early collectors were fully capable of sampling them, thus it is likely that a higher density of these animals now subsist where they were sparse historically.

#### Expectations from species-area theory

Wearn et al. [52] and Rybicki and Hanski [53] developed models to predict “extinction debt,” a measure of impending extinctions based on current habitat degradation, using species-area relationships. Both predict that the reduction of habitat area over time should result in a delayed extinction curve that will begin to be observed approximately 40 years after the initial impact on habitat. Our results compare mammal species richness for two time blocks, each spanning ∼60 years (late 1800's to 1950, 1951-present), thus extinction debt incurred between these two blocks should be detected in our results unless it began since 1950. Comparing 6 currently protected areas with the corresponding larger geographic areas of a century ago, it is clear the area of available minimally disturbed habitat for Kenya's wildlife has decreased [1], [3], [41], [54] (Fig. 1), and many species have been affected by logging, ranching, and other processes within or outside of the protected areas [46], [54]–[55]. Despite these changes, we detect no significant decreases in species richness in the current protected areas (Fig. 3). In fact, the opposite is true for 5 out of the 6 sites, indicating that more species are able to co-exist in smaller protected areas under pressure from habitat loss in their former ranges, albeit with smaller population sizes [1], [10], [32]. Our measures of functional traits (body size distribution and trophic structure) were largely stable over time (Fig. S5), indicating minimal change in community structure even with the addition of new species. The significant body size distribution shifts occurred at the 2 sites with the greatest documented habitat change within the protected area boundaries: Kakamega, exposed to intense human population pressure and logging [3], [41], and Lake Naivasha, which is heavily used by the floriculture and horticulture industries [45].

Our raw data include some species that were recorded in one historical site and no modern sites (Fig. 5A, Table S5 in File S1). Though these may first appear as local extinctions, closer examination reveals this group to be composed of species that (a) are peripheral in their ranges and thus likely to naturally fluctuate in occupancy, (b) are data deficient, (c) have low densities and were less likely to be sampled, (d) have problematic or disputed taxonomy. Only a small group of species appears truly to have been extirpated. Our data cannot detect extinctions or extirpations that occurred after the 1950's and 60's, so it is possible that more extirpations have occurred since then, and Kenya may currently be accumulating extinction debt. However, static or increased richness in most parks demonstrates that species that disappeared were replaced by newly recorded species (Fig. 5B) as well as species increasing their occupancy across protected areas (Fig. 5C) despite the loss of area (Fig. 1). This argues against a decrease in park carrying capacity as the cause of the observed extirpations.

The results of this study contribute new information regarding controls on biodiversity, including species area relationships, the impact of common vs. rare species, and resilience of community structure in the face of environmental change and human impact. Based on our results, richness and diversity patterns in Kenyan protected areas are driven by common, mobile species and by species that are not critically dependent on protected areas to survive. Some of these species exploit niches that may be created by the proximity of human settlements. Our analysis documents an increase in population and cropland density in the immediate vicinity of these protected areas over the last 100 years (Fig. S1). Thus, some components of the mammal communities may not be supported solely by resources within the protected area. Failing to take their mobility and adaptability into account can cause extrapolations based on species area relationships [52]–[53] to underestimate the potential species richness in protected areas.

We cannot rule out the possibility that there is a “community debt” analogous to extinction debt that would allow a community structure to survive for a time even under extreme habitat stress. This concept, comprising measures of community structure (e.g., trophic distribution, body size distribution), could explain why most protected areas are experiencing homeostasis of some aspects of their community composition (e.g., Fig. S5), but also signals those that are showing signs of losing their historic structure (e.g., Kakamega). This is not necessarily an indicator of future collapse, but rather the restructuring of community composition that occurs when human settlement comes into close contact with the edges of a protected area.

## Conclusion

Our study shows that 6 protected areas in Kenya are preserving alpha diversity in their mammal communities while these communities also have become more similar over time. Species able to survive in changing conditions and a range of environments, or those adapted to human-inhabited areas, have expanded their site occupancy, balancing diversity loss in individual sites over the past century. This involves some species turnover but does not affect the mammal diversity of Kenya as a whole and is transforming unique communities into a more homogeneous assemblage of species of all body sizes across a wide geographic area.

Over the past 50+ years, conservation efforts by the Kenyan government have sustained species richness across geographically dispersed protected areas. Though anthropogenic pressure continues to increase, most species historically present in Kenya continue to survive and some have expanded their ranges. Over the same time period that species richness has remained relatively stable, the uniqueness of local mammal communities has declined, primarily as a consequence of this range expansion. Beta diversity contributes to higher regional and continent-scale diversity and ultimately contributes to maintaining species pools on a larger scale. In the future, a stronger focus on conserving beta diversity also could help protect alpha diversity in mammal communities across East Africa.

## Supporting Information

Figure S1 **Anthropogenic habitat alteration in the last 100 years.** Estimates of the population density (A, B) and amount of cropland (C, D) in 1900 (A, C) and 2000 (B, D). The park outlines in both the historical and the modern contain the estimated park area (solid white lines) and a buffer zone (dashed yellow lines). Data on cropland and populations density were taken from HYDE [34]–[35]. Change in color from dark green to red represents change from low to high. The black pixel in panel B represents a population density of 12,000 people/km^2^.
(TIF)

Figure S2 **Population and cropland density around sites, 1900–2000.** Logged estimated population density (A) and amount of cropland (B) in the buffer zones (see Fig. S1) around the protected areas in 1900 (yellow bars) and 2000 (green bars). The increase in population and cropland density in the areas immediately surrounding the parks gives a quantitative estimate of the increase in anthropogenic effects over the last century in Kenya. These data were extracted from the HYDE database [34]–[35].
(TIF)

Figure S3 **Species richness by body size.** Species richness of all species (top left) in historical (yellow) and modern sites (green) and broken down by size class: small (top right), medium (bottom left) and large (bottom right). Note that the increase in richness observed in the majority of the parks is repeated in the different size classes. Only Lake Naivasha shows a decrease in species and this is driven by decreases in small and medium bodied, but not large bodied species. The only other difference is that the number of small-bodied species in Nairobi did not change, and medium and large-bodied species are causing the small increase in richness at that site.
(TIF)

Figure S4 **Beta diversity by size and visibility subset.** Degree of similarity between each pair of sites in the historical and modern records calculated using the Sorensen Index and separated by (A) size class and (B) visibility. Yellow circles indicate similarity between pairs of historical sites, and the green circles indicate similarity between pairs of modern sites. The data show a clear increase in similarity, thus a decrease in beta-diversity, over the past century. Change over time in all panels was highly significant (small: p = 0.0103; medium: p<0.0001; large: p = 0.0006; low visibility: p<0.0001; medium visibility: p = 0.0002; high visibility: p = 0.0006). This is true even when Kakamega, a unique forest affected by deforestation and human population increase over the past century, is excluded from the analyses. Moreover, this holds for large-bodied, high visibility mammals on the lower right. Here, the index increases by small margins, but consistently (missing bubbles indicate no shared species or an index value of 0).
(TIF)

Figure S5 **Body size and trophic distributions.** (A) Body size distributions for each park, comparing historical and modern mammal communities, with significant change observed in Kakamega and Naivasha. (B) Trophic distributions for each park, comparing historical and modern mammal communities. Key: yellow = historic, green = modern, m = meat, p = piscivore, in = invertebrates, ad = animal-dominant omnivore, pd = plant-dominant omnivore, fr = frugivore, b = browser, g = grazer. No significant changes in trophic structure were observed over time. Results of all significance tests for body size and trophic distributions are in Table S6 in File S1.
(TIF)

Figure S6 **Collection curves.** Collection curves for each of the six localities included in this study using specific date information from the Smithsonian African expedition (NMNH; 1909–1911) and the Carl Akeley East Africa Expedition (FMNH; 1905–1906). Breaks of longer than 10 days between collections at a site are excluded from the day count. Day counts may include multiple separate visits. In some cases the curves show several rapid jumps, which correspond to changes in sampling strategy, focus, or season.
(TIF)

Figure S7 **Occupancy vs. population density analysis for small, medium, and large size subclasses.** Blue dots =  Low visibility species, red triangles  =  medium visibility, and black diamonds  =  high visibility. Regressions are in Table S7 in File S1.
(TIF)

Figure S8 **Occupancy vs population density for low, medium, and high visibility subclasses.** Blue dots =  Low visibility species, red triangles  =  medium visibility, and black diamonds  =  high visibility. Regression equations are as follows. Low: y = 0.295–0.066X; r2 = 0.029; F = 0.210; p = 0.661. Medium: y = 0.125+0.028X; r2 = 0.021; F = 0.462; p = 0.504. High: y = 0.094–0.030X; r2 = 0.040; F = 0.883; p = 0.358. All: y = 0.139–0.006X; r2 = 0.0008; F = 0.045; p = 0.834. Dotted line denotes the regression for “All.”
(TIF)

File S1 **Tables S1–S7.** Site information, Occupancy Changes, Jaccard's Values, Full Species Lists, Significance Tests, Regressions.
(DOCX)

File S2 **Appendices A–C: Metadata, Taxonomic Notes, and Site Descriptions.**
(DOCX)
