# Supplementary figures and images for: A Century of Change in Kenya's Mammal Communities: Increased Richness and Decreased Uniqueness in Six Protected Areas
Source: PLoS One. 2014 Apr 9;9(4):e93092. doi: 10.1371/journal.pone.0093092 (PMC3981716; doi:10.1371/journal.pone.0093092)

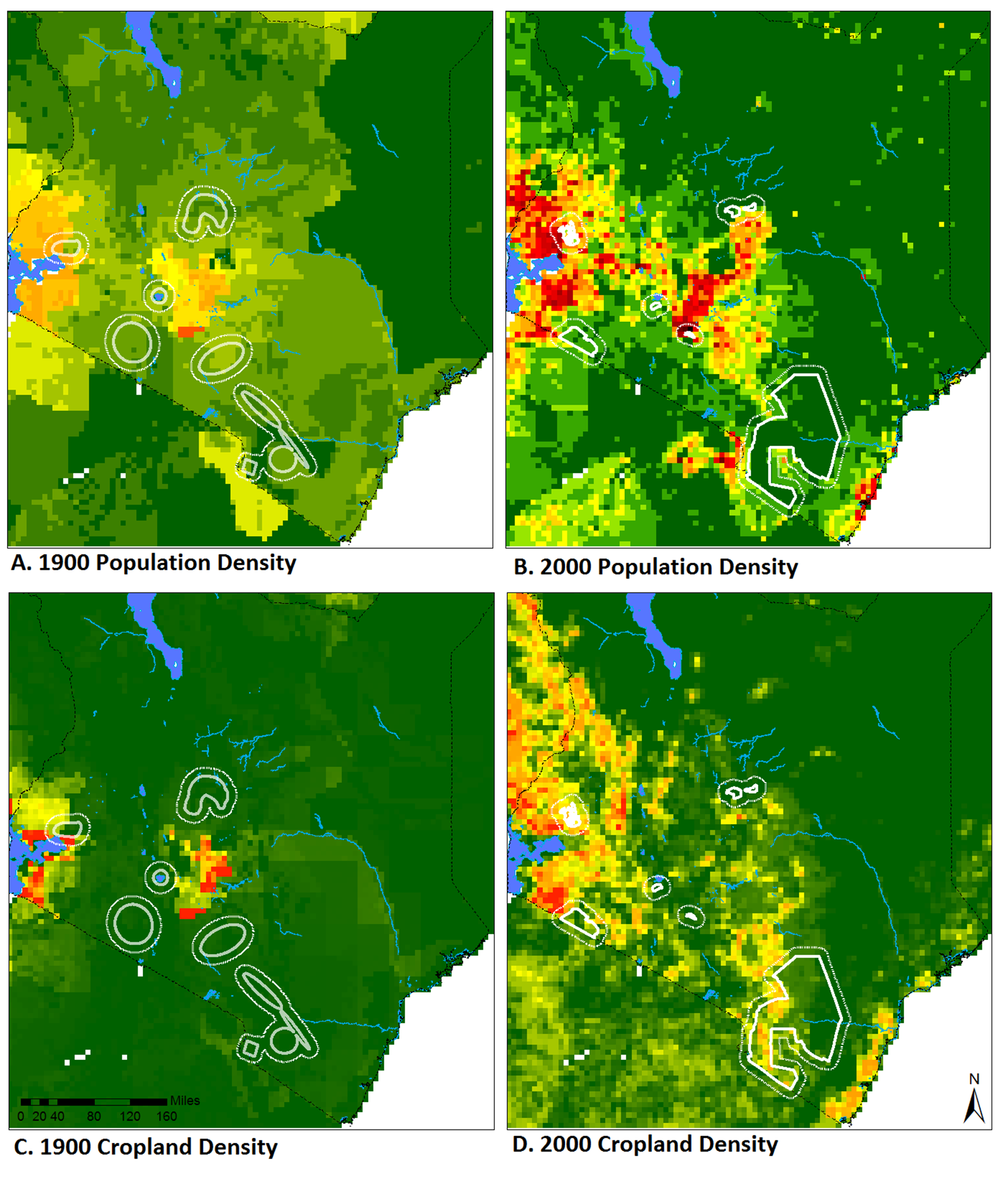

Supplement: Figure S1 — Anthropogenic habitat alteration in the last 100 years. Estimates of the population density (A, B) and amount of cropland (C, D) in 1900 (A, C) and 2000 (B, D). The park outlines in both the historical and the modern contain the estimated park area (solid white lines) and a buffer zone (dashed yellow lines). Data on cropland and populations density were taken from HYDE [34]–[35]. Change in color from dark green to red represents change from low to high. The black pixel in panel B represents a population density of 12,000 people/km2. (TIF) [file pone.0093092.s001.tif]

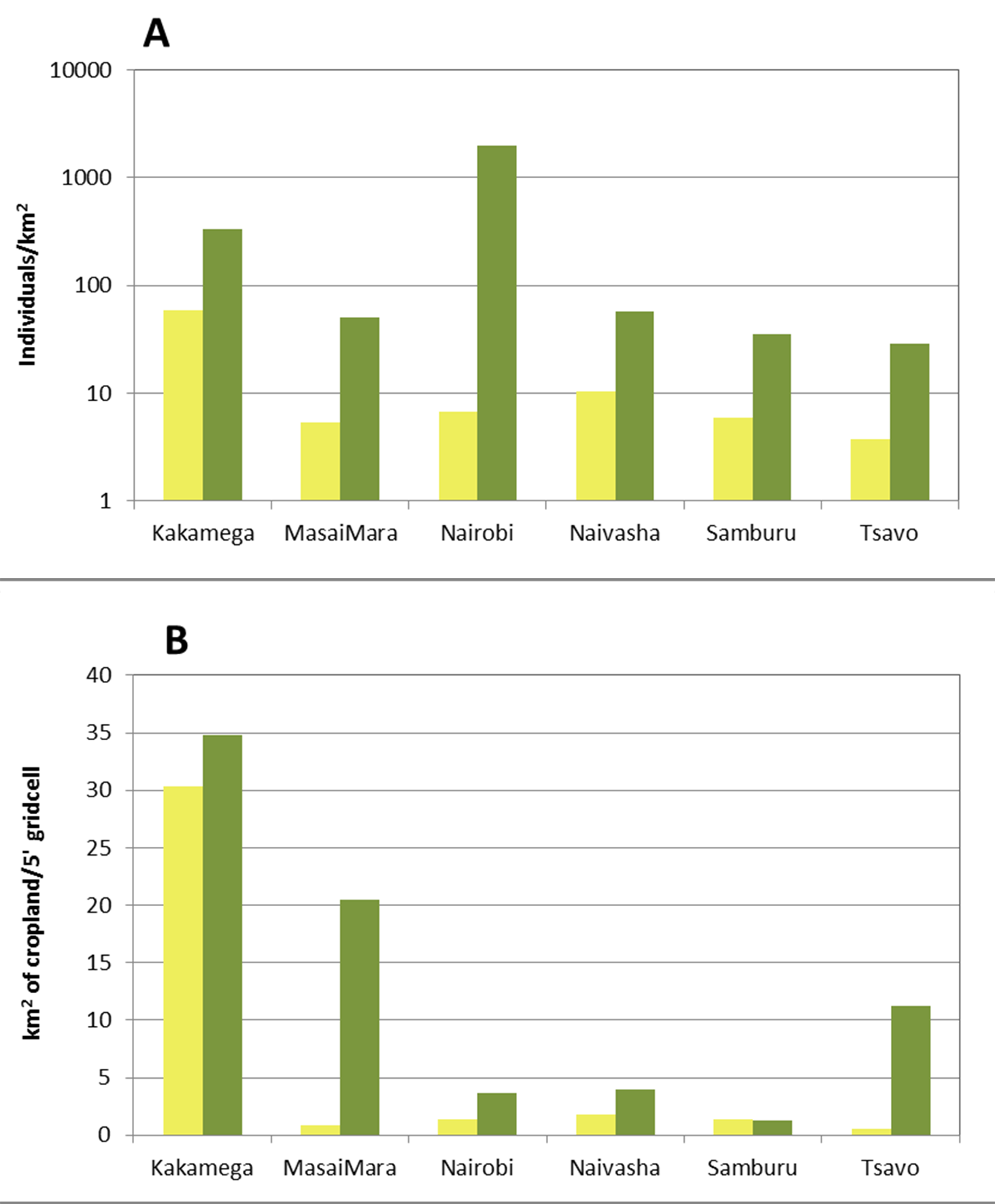

Supplement: Figure S2 — Population and cropland density around sites, 1900–2000. Logged estimated population density (A) and amount of cropland (B) in the buffer zones (see Fig. S1) around the protected areas in 1900 (yellow bars) and 2000 (green bars). The increase in population and cropland density in the areas immediately surrounding the parks gives a quantitative estimate of the increase in anthropogenic effects over the last century in Kenya. These data were extracted from the HYDE database [34]–[35]. (TIF) [file pone.0093092.s002.tif]

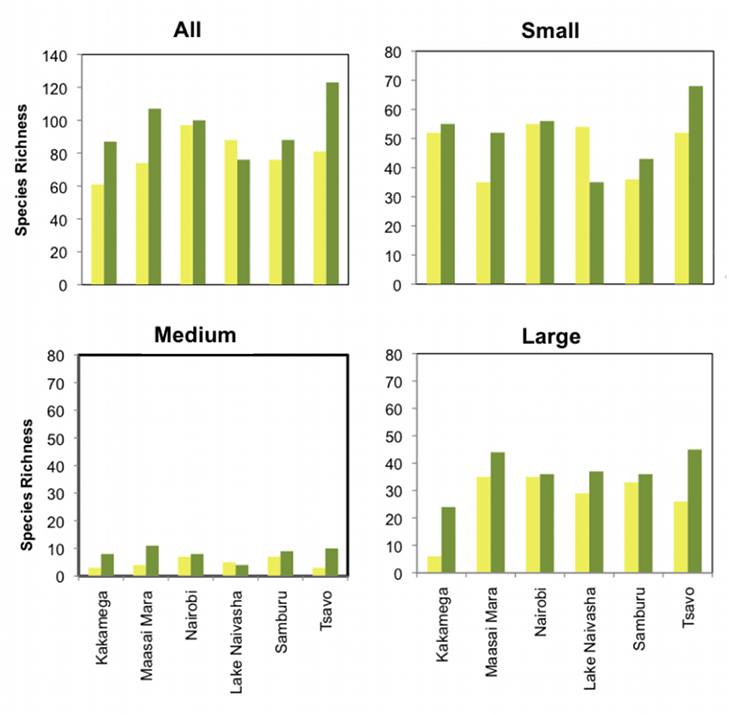

Supplement: Figure S3 — Species richness by body size. Species richness of all species (top left) in historical (yellow) and modern sites (green) and broken down by size class: small (top right), medium (bottom left) and large (bottom right). Note that the increase in richness observed in the majority of the parks is repeated in the different size classes. Only Lake Naivasha shows a decrease in species and this is driven by decreases in small and medium bodied, but not large bodied species. The only other difference is that the number of small-bodied species in Nairobi did not change, and medium and large-bodied species are causing the small increase in richness at that site. (TIF) [file pone.0093092.s003.tif]

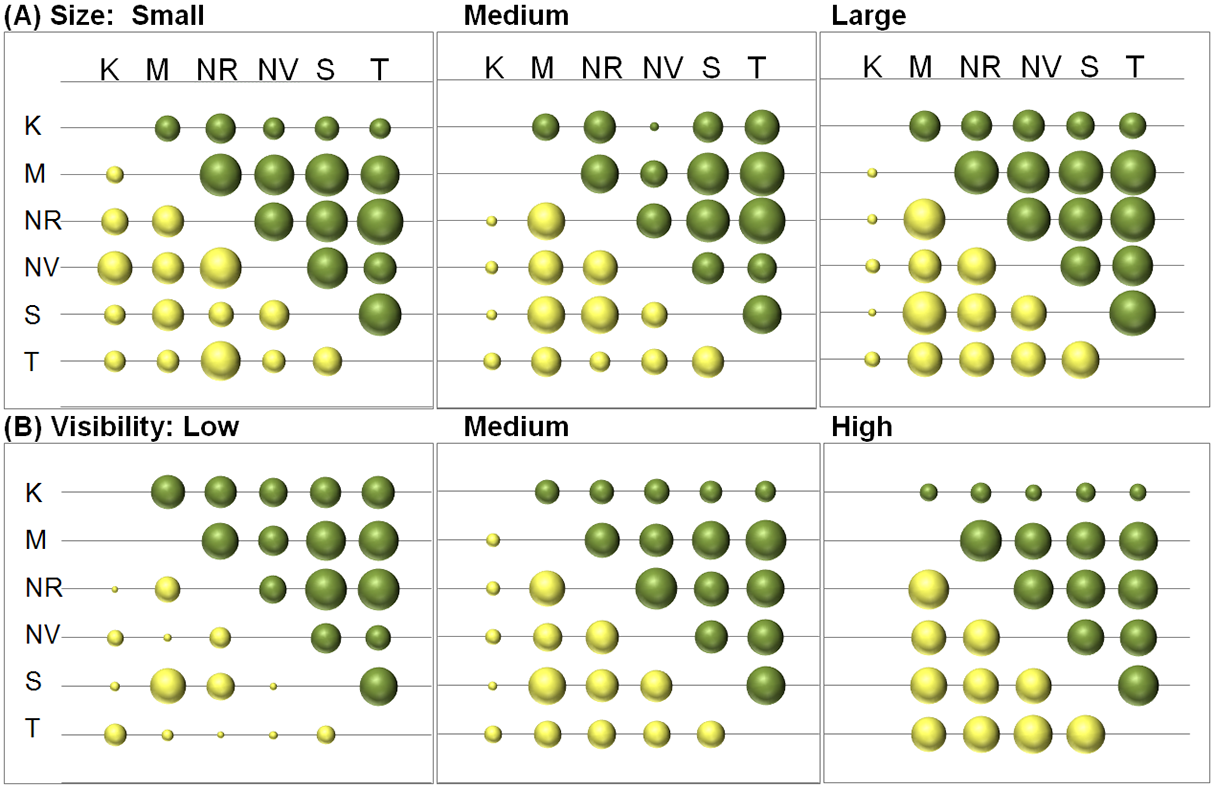

Supplement: Figure S4 — Beta diversity by size and visibility subset. Degree of similarity between each pair of sites in the historical and modern records calculated using the Sorensen Index and separated by (A) size class and (B) visibility. Yellow circles indicate similarity between pairs of historical sites, and the green circles indicate similarity between pairs of modern sites. The data show a clear increase in similarity, thus a decrease in beta-diversity, over the past century. Change over time in all panels was highly significant (small: p = 0.0103; medium: p<0.0001; large: p = 0.0006; low visibility: p<0.0001; medium visibility: p = 0.0002; high visibility: p = 0.0006). This is true even when Kakamega, a unique forest affected by deforestation and human population increase over the past century, is excluded from the analyses. Moreover, this holds for large-bodied, high visibility mammals on the lower right. Here, the index increases by small margins, but consistently (missing bubbles indicate no shared species or an index value of 0). (TIF) [file pone.0093092.s004.tif]

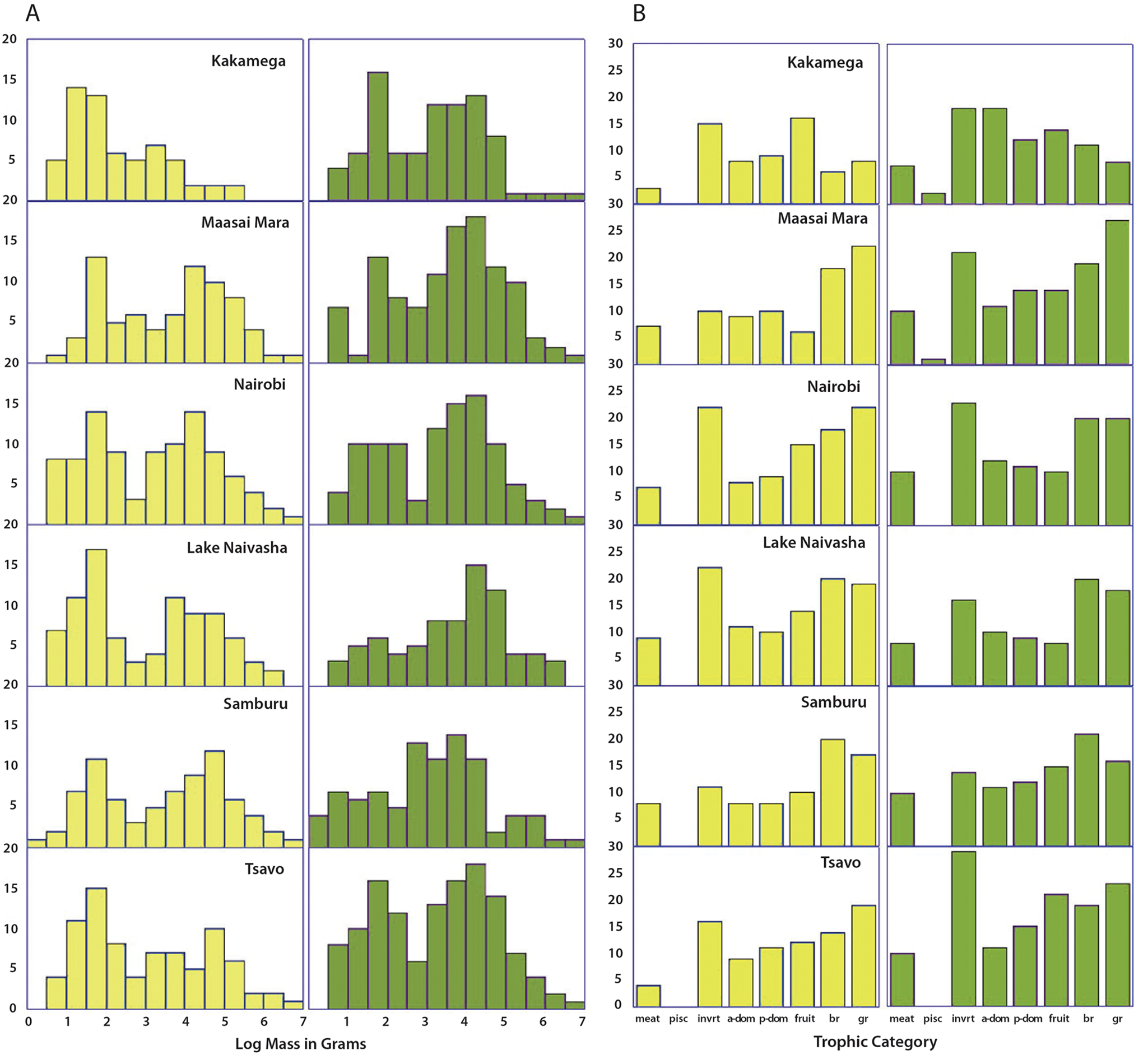

Supplement: Figure S5 — Body size and trophic distributions. (A) Body size distributions for each park, comparing historical and modern mammal communities, with significant change observed in Kakamega and Naivasha. (B) Trophic distributions for each park, comparing historical and modern mammal communities. Key: yellow = historic, green = modern, m = meat, p = piscivore, in = invertebrates, ad = animal-dominant omnivore, pd = plant-dominant omnivore, fr = frugivore, b = browser, g = grazer. No significant changes in trophic structure were observed over time. Results of all significance tests for body size and trophic distributions are in Table S6 in File S1. (TIF) [file pone.0093092.s005.tif]

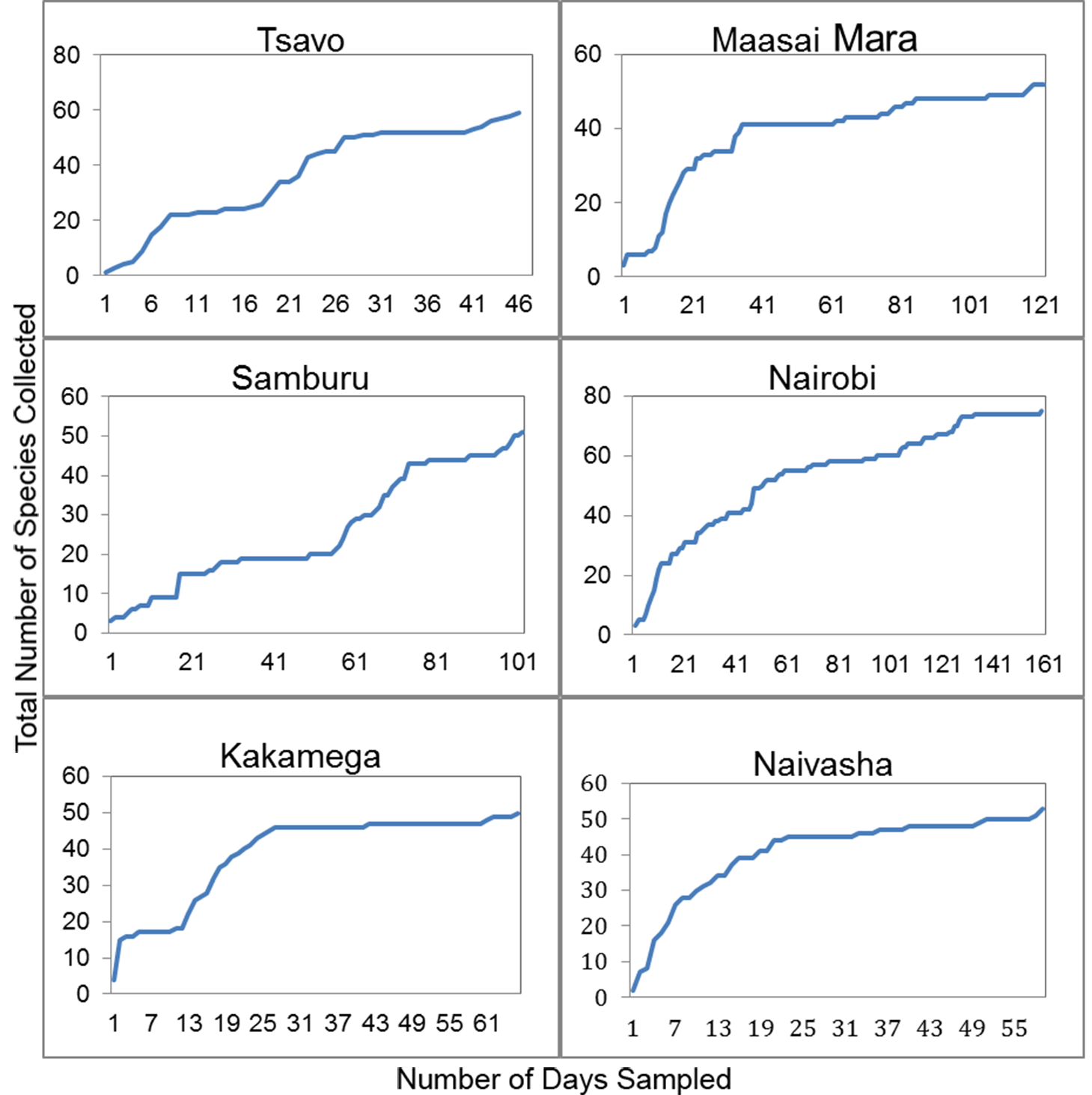

Supplement: Figure S6 — Collection curves. Collection curves for each of the six localities included in this study using specific date information from the Smithsonian African expedition (NMNH; 1909–1911) and the Carl Akeley East Africa Expedition (FMNH; 1905–1906). Breaks of longer than 10 days between collections at a site are excluded from the day count. Day counts may include multiple separate visits. In some cases the curves show several rapid jumps, which correspond to changes in sampling strategy, focus, or season. (TIF) [file pone.0093092.s006.tif]

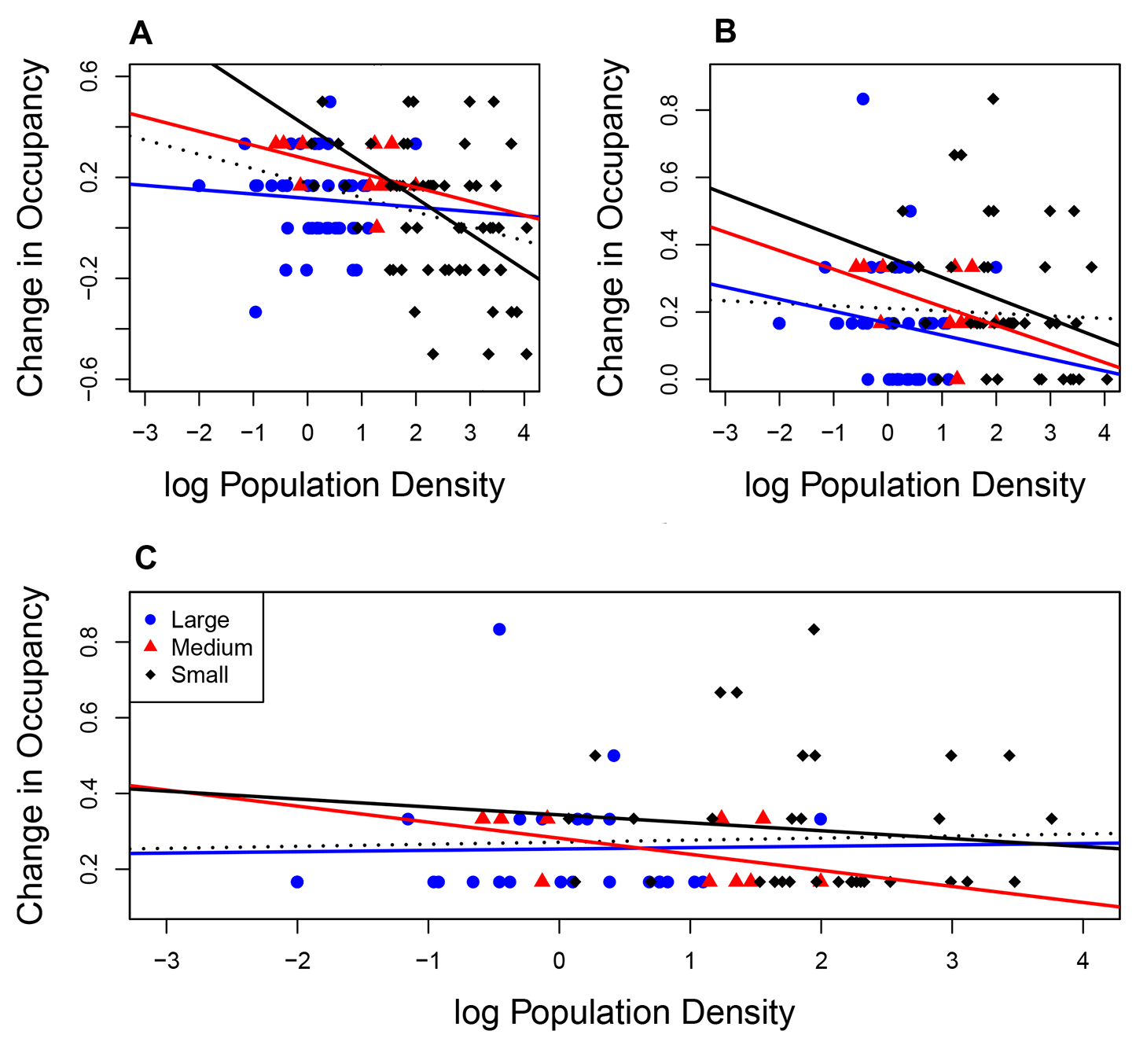

Supplement: Figure S7 — Occupancy vs. population density analysis for small, medium, and large size subclasses. Blue dots = Low visibility species, red triangles = medium visibility, and black diamonds = high visibility. Regressions are in Table S7 in File S1. (TIF) [file pone.0093092.s007.tif]

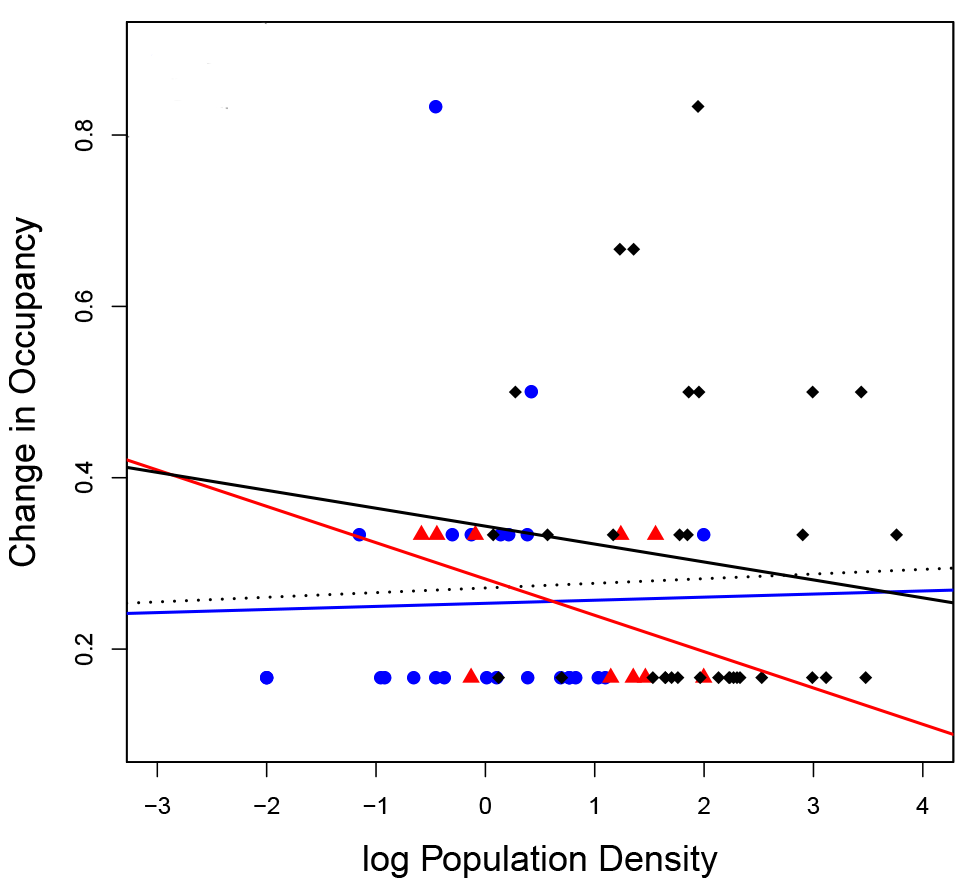

Supplement: Figure S8 — Occupancy vs population density for low, medium, and high visibility subclasses. Blue dots = Low visibility species, red triangles = medium visibility, and black diamonds = high visibility. Regression equations are as follows. Low: y = 0.295–0.066X; r2 = 0.029; F = 0.210; p = 0.661. Medium: y = 0.125+0.028X; r2 = 0.021; F = 0.462; p = 0.504. High: y = 0.094–0.030X; r2 = 0.040; F = 0.883; p = 0.358. All: y = 0.139–0.006X; r2 = 0.0008; F = 0.045; p = 0.834. Dotted line denotes the regression for “All.” (TIF) [file pone.0093092.s008.tif]
