# Supplementary material for: A Century of Change in Kenya's Mammal Communities: Increased Richness and Decreased Uniqueness in Six Protected Areas
Source: PLoS One. 2014 Apr 9;9(4):e93092. doi: 10.1371/journal.pone.0093092 (PMC3981716; doi:10.1371/journal.pone.0093092)
Supplement: File S1 — Tables S1–S7. Site information, Occupancy Changes, Jaccard's Values, Full Species Lists, Significance Tests, Regressions. (DOCX) [file pone.0093092.s009.docx]

**Data Tables**

| **Site** | **Modern Area (km^2^)** | **Estimated Hist. Area (km^2^)** | **Sampling Effort (Days)** | **Number of Species (Expedition Specimens Only)** | **Specimens Collected** |
| --- | --- | --- | --- | --- | --- |
| **Kakamega** | 540 | 800 | 67 | 50 | 769 |
| **Maasai Mara** | 1510 | 1300 | 122 | 52 | 293 |
| **Nairobi** | 120 | 700 | 160 | 75 | 776 |
| **Naivasha** | 200 | 800 | 59 | 53 | 549 |
| **Samburu** | 430 | 2500 | 102 | 51 | 212 |
| **Tsavo** | 20,000 | 4000 | 46 | 59 | 421 |

**Table S1.** **Collection information** from the Smithsonian African expedition (NMNH; 1909-1910) and the Carl Akeley East Africa Expedition (FMNH; 1905-1906) including the modern site sizes (including adjacent parks included in data collection), estimated area for each historical site, the number of days researchers collected in each area, the number of species and specimens collected [1].

| **(A)Weights** | | | | | | | |
| --- | --- | --- | --- | --- | --- | --- | --- |
|  | **Initial Occupancy** | | | | | | |
| **Change in Occupancy** | **0** | **1** | **2** | **3** | **4** | **5** | **6** |
| **6** | 0 |  |  |  |  |  |  |
| **5** | -5 | 5 |  |  |  |  |  |
| **4** | -8 | 0 | 8 |  |  |  |  |
| **3** | -9 | -3 | 3 | 9 |  |  |  |
| **2** | -8 | -4 | 0 | 4 | 8 |  |  |
| **1** | -5 | -3 | -1 | 1 | 3 | 5 |  |
| **0** | **0** | **0** | **0** | **0** | **0** | **0** | **0** |
| **-1** |  | 5 | 3 | 1 | -1 | -3 | -5 |
| **-2** |  |  | 8 | 4 | 0 | -4 | -8 |
| **-3** |  |  |  | 9 | 3 | -3 | -9 |
| **-4** |  |  |  |  | 8 | 0 | -8 |
| **-5** |  |  |  |  |  | 5 | -5 |
| **-6** |  |  |  |  |  |  | 0 |
| **(B) Number of Species Exhibiting Each Occupancy Change** | | | | | | | |
| **6** | 0 |  |  |  |  |  |  |
| **5** | 1 | 1 |  |  |  |  |  |
| **4** | 1 | 1 | 2 |  |  |  |  |
| **3** | 3 | 1 | 4 | 6 |  |  |  |
| **2** | 9 | 5 | 3 | 9 | 7 |  |  |
| **1** | 38 | 11 | 7 | 4 | 9 | 6 |  |
| **0** |  | 20 | 8 | 4 | 2 | 7 | 1 |
| **-1** |  | 30 | 12 | 5 | 3 | 1 | 1 |
| **-2** |  |  | 5 | 4 | 2 | 3 | 1 |
| **-3** |  |  |  | 2 | 3 | 0 | 1 |
| **-4** |  |  |  |  | 1 | 1 | 0 |
| **-5** |  |  |  |  |  | 0 | 0 |
| **-6** |  |  |  |  |  |  | 0 |
| **(C) Product of A and B** | | | | | | | |
| **6** | 0 |  |  |  |  |  |  |
| **5** | -5 | 5 |  |  |  |  |  |
| **4** | -8 | 0 | 16* |  |  |  |  |
| **3** | -27 | -3 | 12* | 54* |  |  |  |
| **2** | -72 | -20 | 0 | 36* | 56* |  |  |
| **1** | -190 | -33 | -7 | 4 | 27* | 30* |  |
| **0** | 0 | 0 | 0 | 0 | 0 | 0 | 0 |
| **-1** |  | 150 | 36 | 5 | -3 | -3 | -5 |
| **-2** |  |  | 40 | 16 | 0 | -12 | -8 |
| **-3** |  |  |  | 18 | 9 | 0 | -9 |
| **-4** |  |  |  |  | 8 | 0 | 0 |
| **-5** |  |  |  |  |  | 0 | 0 |
| **-6** |  |  |  |  |  |  | 0 |

**Table S2**. **Assessment of the effect of the occupancy changes of species**. (A) Weights for each possible change in occupancy, based on net change in species overlap in paired comparisons out of 15 total pairs. (B) Total number of species exhibiting each occupancy change. (C) Product of (A) and (B), showing net effect of occupancy changes. *Categories that are driving the pattern after the changes in the occupancies of rare species have canceled each other out.

| **Historic** | | **All** | **Small** | **Med** | **Large** | **All L&M** | **Low Vis** | **Med Vis** | **High Vis** |
| --- | --- | --- | --- | --- | --- | --- | --- | --- | --- |
| K | MM | 0.207 | 0.230 | 0.000 | 0.195 | 0.060 | 0.000 | 0.286 | 0.000 |
| K | NR | 0.304 | 0.355 | 0.200 | 0.195 | 0.078 | 0.143 | 0.300 | 0.000 |
| K | NV | 0.403 | 0.453 | 0.250 | 0.286 | 0.104 | 0.364 | 0.333 | 0.000 |
| K | S | 0.234 | 0.273 | 0.200 | 0.154 | 0.059 | 0.200 | 0.190 | 0.000 |
| K | T | 0.296 | 0.288 | 0.333 | 0.313 | 0.110 | 0.500 | 0.375 | 0.000 |
| MM | NR | 0.608 | 0.422 | 0.727 | 0.829 | 0.552 | 0.571 | 0.774 | 0.889 |
| MM | NV | 0.531 | 0.427 | 0.667 | 0.656 | 0.422 | 0.182 | 0.621 | 0.765 |
| MM | S | 0.640 | 0.423 | 0.727 | 0.853 | 0.561 | 0.800 | 0.813 | 0.811 |
| MM | T | 0.465 | 0.299 | 0.571 | 0.689 | 0.423 | 0.250 | 0.593 | 0.765 |
| NR | NV | 0.627 | 0.550 | 0.667 | 0.750 | 0.409 | 0.471 | 0.714 | 0.813 |
| NR | S | 0.532 | 0.330 | 0.714 | 0.765 | 0.438 | 0.625 | 0.710 | 0.800 |
| NR | T | 0.573 | 0.523 | 0.400 | 0.689 | 0.346 | 0.143 | 0.615 | 0.813 |
| NV | S | 0.524 | 0.400 | 0.500 | 0.710 | 0.375 | 0.154 | 0.690 | 0.788 |
| NV | T | 0.438 | 0.302 | 0.500 | 0.691 | 0.356 | 0.182 | 0.583 | 0.867 |
| S | T | 0.535 | 0.386 | 0.600 | 0.746 | 0.453 | 0.400 | 0.593 | 0.848 |
| **MEAN** | | 0.461 | 0.377 | 0.470 | 0.568 | 0.316 | 0.332 | 0.546 | 0.544 |
| **Modern** | | **All** | **Small** | **Med** | **Large** | **All L&M** | **Low Vis** | **Med Vis** | **High Vis** |
| K | MM | 0.454 | 0.336 | 0.526 | 0.618 | 0.352 | 0.759 | 0.529 | 0.400 |
| K | NR | 0.481 | 0.396 | 0.625 | 0.600 | 0.331 | 0.714 | 0.519 | 0.455 |
| K | NV | 0.405 | 0.289 | 0.167 | 0.623 | 0.349 | 0.640 | 0.538 | 0.364 |
| K | S | 0.434 | 0.327 | 0.588 | 0.567 | 0.344 | 0.692 | 0.483 | 0.435 |
| K | T | 0.390 | 0.276 | 0.667 | 0.522 | 0.289 | 0.733 | 0.452 | 0.370 |
| MM | NR | 0.696 | 0.556 | 0.737 | 0.875 | 0.535 | 0.815 | 0.757 | 0.919 |
| MM | NV | 0.667 | 0.529 | 0.533 | 0.840 | 0.541 | 0.667 | 0.722 | 0.811 |
| MM | S | 0.718 | 0.568 | 0.800 | 0.875 | 0.589 | 0.880 | 0.821 | 0.842 |
| MM | T | 0.696 | 0.517 | 0.857 | 0.899 | 0.536 | 0.897 | 0.878 | 0.857 |
| NR | NV | 0.670 | 0.505 | 0.667 | 0.877 | 0.574 | 0.609 | 0.897 | 0.882 |
| NR | S | 0.691 | 0.545 | 0.824 | 0.861 | 0.563 | 0.917 | 0.750 | 0.857 |
| NR | T | 0.744 | 0.613 | 0.889 | 0.889 | 0.512 | 0.929 | 0.824 | 0.872 |
| NV | S | 0.659 | 0.538 | 0.615 | 0.795 | 0.489 | 0.667 | 0.710 | 0.800 |
| NV | T | 0.593 | 0.427 | 0.571 | 0.805 | 0.429 | 0.560 | 0.788 | 0.821 |
| S | T | 0.711 | 0.559 | 0.737 | 0.914 | 0.509 | 0.846 | 0.833 | 0.900 |
| **MEAN** | | 0.601 | 0.465 | 0.654 | 0.770 | 0.463 | 0.755 | 0.700 | 0.706 |

**Table S3**. **Sorensen index values for size and visibility classes across space**. Assuming species turnover is proportional to the inverse of similarity, the spatial turnover among modern sites is barely greater than the temporal turnover from historical to modern sites, dropping below it for large mammals. The spatial turnover in historic Kenya, however, is much greater than the temporal turnover. Thus, the average modern site is about as similar to another modern site as a historical site is to the same site now, whereas historic sites were much more different from each other. Key: Small = 0-5kg, Med = 5-10kg, Large >10kg). Right side panels are Large and Medium sized mammals only, divided by visibility class (low, medium, and high). K = Kakamega, MM = Masaai Mara, NR = Nairobi, NV = Naivasha, S = Samburu, T = Tsavo.

|  | **All** | **Small** | **Med** | **Large** |
| --- | --- | --- | --- | --- |
| **Kenya (6 sites)** | 0.780 | 0.713 | 0.870 | 0.933 |
| **Kakamega** | 0.486 | 0.523 | 0.545 | 0.333 |
| **Maasai Mara** | 0.641 | 0.483 | 0.571 | 0.825 |
| **Nairobi** | 0.722 | 0.595 | 0.857 | 0.873 |
| **Lake Naivasha** | 0.598 | 0.472 | 0.667 | 0.758 |
| **Samburu** | 0.720 | 0.557 | 0.875 | 0.870 |
| **Tsavo** | 0.539 | 0.433 | 0.462 | 0.732 |
| **MEAN** | 0.641 | 0.539 | 0.692 | 0.761 |

**Table S4.** **Sorensen index values for each site over time**. If the species turnover is proportional to the inverse of similarity, note that the spatial turnover (Table S7) is barely greater than the temporal turnover in the modern sites, dropping below it for large mammals. The spatial turnover in historic Kenya, however, is much greater than the temporal turnover. This means that the average modern site is about as similar to another modern site as a historical site is to the same site presently, whereas historic sites were much more diverse. Columns include all mammals, small mammals (0-5kg), medium mammals (5-10kg), and large mammals (>10kg), respectively.

|  |  | KK | | MM | | NR | | NV | | SB | | TV | |
| --- | --- | --- | --- | --- | --- | --- | --- | --- | --- | --- | --- | --- | --- |
| Size | Scientific Name | H | M | H | M | H | M | H | M | H | M | H | M |
| Lg | *Acinonyx jubatus* |  |  | x | x | x | x |  | x | x | x | x | x |
|  | *Aepyceros melampus* |  |  | x | x | x | x | x | x | x | x | x | x |
|  | *Alcelaphus buselaphus* |  |  | x | x | x | x | x | x |  |  | x | x |
|  | *Aonyx capensis* |  | x |  | x |  | x | x | x |  | x |  | x |
|  | *Beatragus hunteri* |  |  |  |  |  |  |  |  |  |  |  | x |
|  | *Canis adustus* | x | x | x | x | x | x |  | x |  | x | x | x |
|  | *Canis aureus* |  |  | x | x |  |  | x | x | x | x |  | x |
|  | *Caracal caracal* |  |  |  | x |  | x |  |  |  | x |  | x |
|  | *Cephalophus harveyi* |  |  |  |  | x | x |  |  |  |  | x | x |
|  | *Cephalophus silvicultor* |  | x |  |  |  |  |  | x |  |  |  |  |
|  | *Cephalophus weynsi* |  | x |  |  | x |  |  |  |  |  |  |  |
|  | *Civettictis civetta* | x | x |  | x |  | x | x | x |  | x | x | x |
|  | *Colobus guereza* |  | x |  | x | x | x | x | x |  |  |  |  |
|  | *Connochaetes taurinus* |  |  | x | x | x | x | x | x | x |  | x | x |
|  | *Crocuta crocuta* |  | x | x | x | x | x | x | x | x | x | x | x |
|  | *Damaliscus korrigum* |  |  | x | x |  |  |  |  |  |  |  |  |
|  | *Diceros bicornis* |  | x | x | x | x | x | x | x | x | x | x | x |
|  | *Ceratotherium simum* |  |  |  | x |  |  |  | x |  |  |  |  |
|  | *Equus burchellii* |  |  | x | x | x | x | x | x | x | x | x | x |
|  | *Equus grevyi* |  |  |  |  |  |  |  |  | x | x |  | x |
|  | *Eudorcas thomsonii* |  |  | x | x | x | x | x | x | x | x |  | x |
|  | *Giraffa camelopardalis* |  |  | x | x | x | x | x | x | x | x | x | x |
|  | *Hippopotamus amphibius* |  |  |  | x | x | x | x | x | x | x | x | x |
|  | *Hippotragus equinus* |  |  | x | x | x |  |  |  |  |  |  |  |
|  | *Hyaena hyaena* |  |  | x | x |  | x |  | x | x | x |  | x |
|  | *Hylochoerus meinertzhageni* | x | x |  | x |  |  | x |  |  |  |  |  |
|  | *Hystrix africaeaustralis* |  | x |  | x |  |  |  | x |  |  |  |  |
|  | *Hystrix cristata* |  | x |  | x | x | x | x | x |  | x |  | x |
|  | *Kobus ellipsiprymnus* |  |  | x | x | x | x | x | x | x | x | x | x |
|  | *Leptailurus serval* |  | x | x | x | x | x |  | x | x | x |  | x |
|  | *Litocranius walleri* |  |  |  |  |  |  | x | x | x | x | x | x |
|  | *Loxodonta africana* |  | x | x | x | x | x |  |  | x | x | x | x |
|  | *Lycaon pictus* |  |  | x | x | x | x | x |  |  | x |  | x |
|  | *Nanger granti* |  |  | x | x | x | x | x | x | x | x | x | x |
|  | *Oreotragus oreotragus* |  | x | x | x | x | x |  | x | x | x |  | x |
|  | *Orycteropus afer* |  | x | x | x | x | x | x | x | x | x |  | x |
|  | *Oryx beisa* |  |  |  | x |  |  |  |  | x | x |  | x |
|  | *Ourebia ourebi* |  |  | x | x |  |  |  |  |  |  |  | x |
|  | *Panthera leo* | x |  | x | x | x | x | x |  | x | x | x | x |
|  | *Panthera pardus* |  | x | x | x | x | x | x | x | x | x |  | x |
|  | *Papio anubis* |  | x | x | x | x | x | x | x | x | x | x | x |
|  | *Papio cynocephalus* |  |  |  |  |  |  |  |  |  |  |  | x |
|  | *Phacochoerus africanus* |  | x | x | x |  | x | x | x | x | x | x | x |
|  | *Potamochoerus larvatus* |  | x |  | x | x |  | x |  | x |  | x | x |
|  | *Profelis aurata* |  | x |  |  |  |  |  |  |  |  |  |  |
|  | *Raphicerus campestris* |  |  | x | x | x | x | x | x | x | x |  | x |
|  | *Redunca fulvorufula* |  |  | x |  | x | x |  | x | x |  |  | x |
|  | *Redunca redunca* |  |  | x | x | x | x |  | x |  |  | x | x |
|  | *Sylvicapra grimmia* |  | x | x | x | x | x |  | x | x | x | x | x |
|  | *Syncerus caffer* |  | x | x | x | x | x | x | x | x | x | x | x |
|  | *Taurotragus oryx* |  |  | x | x | x | x |  | x | x | x |  | x |
|  | *Tragelaphus eurycerus* |  |  |  |  | x |  | x | x |  |  |  |  |
|  | *Tragelaphus imberbis* |  |  |  | x |  |  |  |  | x | x | x | x |
|  | *Tragelaphus scriptus* | x | x | x | x | x | x | x | x | x | x | x | x |
|  |  | KK | | MM | | NR | | NV | | SB | | TV | |
| Size | Scientific Name | H | M | H | M | H | M | H | M | H | M | H | M |
| Lg | *Tragelaphus spekii* | x | x |  |  |  |  |  |  |  |  |  |  |
|  | *Tragelaphus strepsiceros* |  |  | x | x |  |  |  |  | x | x | x | x |
| Lg Total | | 6 | 24 | 35 | 44 | 35 | 36 | 29 | 37 | 33 | 36 | 26 | 45 |
| Md | *Canis mesomelas* |  |  | x | x | x | x | x | x | x | x |  | x |
|  | *Cercopithecus neglectus* |  | x |  |  |  |  |  |  |  |  |  |  |
|  | *Chlorocebus pygerythrus* |  | x | x | x | x | x | x | x | x | x | x | x |
|  | *Chlorocebus tantalus* |  |  |  |  | x |  |  |  |  |  |  |  |
|  | *Erythrocebus patas* |  |  |  | x |  |  |  |  |  | x |  |  |
|  | *Felis silvestris* | x | x |  | x |  | x |  |  | x | x | x | x |
|  | *Herpestes ichneumon* | x | x |  | x | x | x | x |  |  | x |  | x |
|  | *Madoqua guentheri* |  | x |  |  |  |  |  |  | x | x |  |  |
|  | *Madoqua kirkii* |  |  | x | x | x | x | x | x | x | x | x | x |
|  | *Manis temminckii* |  |  |  | x |  |  |  |  |  |  |  | x |
|  | *Mellivora capensis* |  | x |  | x | x | x |  |  | x | x |  | x |
|  | *Neotragus moschatus* |  | x |  |  |  | x | x |  |  |  |  | x |
|  | *Philantomba monticola* | x | x |  | x |  |  |  |  |  |  |  | x |
|  | *Proteles cristata* |  |  | x | x | x | x |  | x | x | x |  | x |
| Md Total | | 3 | 8 | 4 | 11 | 7 | 8 | 5 | 4 | 7 | 9 | 3 | 10 |
| Sm | *Acomys cahirinus* |  |  |  |  |  |  |  |  | x |  |  | x |
|  | *Acomys ignitus* |  |  |  |  |  |  |  |  |  |  | x | x |
|  | *Acomys kempi* |  |  | x |  |  | x |  |  | x | x |  |  |
|  | *Acomys percivali* |  |  |  |  |  |  |  |  | x |  |  |  |
|  | *Acomys spp.* |  |  |  |  |  |  |  |  |  |  | x |  |
|  | *Acomys wilsoni* |  |  |  |  |  | x |  |  | x | x | x | x |
|  | *Aethomys chrysophilus* |  |  |  |  |  |  |  | x |  |  | x |  |
|  | *Aethomys hindei* | x | x |  |  | x |  |  |  | x |  | x |  |
|  | *Aethomys kaiseri* | x |  | x | x | x |  |  |  |  |  | x |  |
|  | *Aethomys spp.* |  |  |  |  |  |  |  |  |  |  |  | x |
|  | *Anomalurus derbianus* |  | x |  |  |  |  |  |  |  |  |  |  |
|  | *Arvicanthis abyssinicus* |  |  |  |  | x |  | x |  |  |  | x |  |
|  | *Arvicanthis nairobae* |  |  | x | x | x | x |  |  |  |  |  | x |
|  | *Arvicanthis neumanni* | x |  |  |  |  |  |  |  | x | x |  |  |
|  | *Arvicanthis niloticus* | x | x | x | x | x |  | x |  | x | x |  |  |
|  | *Atelerix albiventris* |  | x | x | x | x | x |  |  | x | x | x | x |
|  | *Atherurus africanus* | x | x |  |  |  |  |  |  |  |  |  |  |
|  | *Atilax paludinosus* |  | x |  | x | x | x | x | x |  | x | x | x |
|  | *Bdeogale crassicauda* |  |  |  |  |  | x |  |  |  |  |  | x |
|  | *Bdeogale jacksoni* |  | x |  |  |  |  |  |  |  |  |  |  |
|  | *Bdeogale nigripes* |  | x |  |  |  |  |  |  |  |  |  |  |
|  | *Beamys hindei* |  |  |  |  |  |  |  |  |  |  |  | x |
|  | *Beamys major* |  |  |  |  |  |  |  |  |  |  |  | x |
|  | *Cercopithecus ascanius* | x | x |  |  |  |  |  |  |  |  |  |  |
|  | *Cercopithecus mitis* |  | x | x | x | x | x | x | x |  | x | x | x |
|  | *Colomys goslingi* |  | x |  |  |  |  |  |  |  |  |  |  |
|  | *Cricetomys ansorgei* | x |  |  |  |  | x |  |  |  | x | x | x |
|  | *Cricetomys gambianus* |  |  |  |  | x | x |  |  |  |  |  | x |
|  | *Crocidura allex* |  |  |  |  |  |  | x |  |  |  |  |  |
|  | *Crocidura attila* |  |  |  |  |  | x |  | x |  |  |  |  |
|  | *Crocidura cyanea* |  |  |  | x |  |  |  |  |  |  | x |  |
|  | *Crocidura elgonius* | x | x |  |  |  |  |  |  |  |  |  |  |
|  | *Crocidura fischeri* |  |  |  |  | x |  |  |  |  |  |  |  |
|  | *Crocidura flavescens* |  | x |  |  |  |  |  |  |  |  |  |  |
|  | *Crocidura fulvastra* |  |  |  |  |  |  |  |  |  |  | x |  |
|  | *Crocidura fumosa* | x | x |  |  |  | x | x |  |  |  |  |  |
|  | *Crocidura fuscomurina* |  |  |  |  | x | x |  |  |  |  |  | x |
|  |  | KK | | MM | | NR | | NV | | SB | | TV | |
| Size | Scientific Name | H | M | H | M | H | M | H | M | H | M | H | M |
| Sm | *Crocidura gracilipes* |  |  | x |  | x |  | x | x |  |  | x |  |
|  | *Crocidura hildegardeae* |  |  |  |  |  |  | x |  | x |  |  | x |
|  | *Crocidura hirta* |  |  |  | x | x | x |  |  |  |  |  |  |
|  | *Crocidura jacksoni* | x |  |  | x | x | x | x |  | x |  | x | x |
|  | *Crocidura littoralis* | x |  |  |  |  |  |  |  |  |  |  |  |
|  | *Crocidura luna* | x |  |  |  | x | x | x |  |  |  |  | x |
|  | *Crocidura maurisca* | x |  |  |  |  |  |  |  |  |  |  |  |
|  | *Crocidura monax* |  | x |  |  |  |  |  |  |  |  |  |  |
|  | *Crocidura montis* |  |  |  |  |  |  | x |  |  |  |  |  |
|  | *Crocidura nigricans* |  |  |  |  | x |  |  |  |  |  |  |  |
|  | *Crocidura nigrofusca* | x | x |  |  |  |  | x |  | x |  | x | x |
|  | *Crocidura olivieri* | x | x |  | x |  | x | x |  |  |  |  | x |
|  | *Crocidura parvipes* |  |  |  |  |  | x |  |  |  |  | x |  |
|  | *Crocidura selina* | x |  |  |  |  |  |  |  |  |  |  | x |
|  | *Crocidura spp.* |  | x |  | x |  |  |  |  |  |  |  |  |
|  | *Crocidura turba* | x |  |  |  | x |  | x |  |  |  |  | x |
|  | *Crocidura viaria* |  |  |  |  | x | x |  |  |  |  |  | x |
|  | *Crocidura voi* |  |  |  |  |  |  |  |  | x |  | x |  |
|  | *Crocidura zaphiri* | x |  |  |  |  |  |  |  |  |  |  |  |
|  | *Dasymys incomtus* | x |  |  |  | x |  | x |  | x | x | x |  |
|  | *Dendrohyrax arboreus* |  | x | x | x |  | x | x | x |  | x |  | x |
|  | *Dendromus insignis* | x |  |  |  |  | x | x |  |  |  |  | x |
|  | *Dendromus melanotis* |  |  |  |  | x |  |  |  |  |  |  |  |
|  | *Dendromus mesomelas* | x |  |  |  |  |  |  |  |  |  |  |  |
|  | *Dendromus messorius* | x |  |  |  |  |  |  |  |  |  |  |  |
|  | *Dendromus mysticalis* | x |  |  | x | x |  | x |  |  |  |  | x |
|  | *Dipodillus harwoodi* |  |  | x |  |  |  | x |  |  |  |  |  |
|  | *Elephantulus brachyrhynchus* |  |  | x |  | x | x | x |  |  |  |  | x |
|  | *Elephantulus rufescens* |  |  | x | x |  |  |  | x | x | x | x | x |
|  | *Galago demidoff* |  | x |  |  |  |  |  |  |  |  |  |  |
|  | *Galago moholi* |  | x |  |  |  |  |  |  |  |  |  |  |
|  | *Galago senegalensis* |  | x | x | x | x | x |  | x |  | x | x | x |
|  | *Galago zanzibaricus* |  |  |  |  |  |  |  |  |  |  |  | x |
|  | *Galerella sanguinea* | x | x | x | x | x | x | x | x |  | x | x | x |
|  | *Genetta genetta* |  | x |  | x | x | x |  | x | x | x | x | x |
|  | *Genetta maculata* | x | x |  |  | x | x | x |  | x | x | x |  |
|  | *Genetta servalina* | x | x |  |  |  |  |  |  |  |  |  |  |
|  | *Genetta tigrina* |  |  |  | x | x | x |  | x |  | x | x | x |
|  | *Gerbilliscus boehmi* |  |  | x | x |  |  |  |  |  |  |  |  |
|  | *Gerbilliscus kempi* | x |  |  |  |  |  |  |  |  |  |  |  |
|  | *Gerbilliscus nigricaudus* |  |  |  |  |  |  |  |  | x |  | x | x |
|  | *Gerbilliscus robustus* |  |  |  |  | x |  |  |  | x |  | x |  |
|  | *Gerbilliscus spp.* |  |  |  |  |  |  |  |  |  | x |  | x |
|  | *Gerbillus pusillus* |  |  |  |  |  |  |  |  |  |  | x |  |
|  | *Gerbillus spp.* |  |  |  |  |  |  |  |  |  |  | x |  |
|  | *Grammomys cometes* | x | x |  |  |  |  |  |  |  |  |  |  |
|  | *Grammomys dolichurus* | x | x | x |  | x | x | x |  | x |  | x | x |
|  | *Grammomys ibeanus* |  |  |  |  |  |  | x |  | x | x |  |  |
|  | *Grammomys macmillani* |  |  |  |  |  |  |  |  |  |  | x | x |
|  | *Grammomys spp.* |  |  |  |  |  |  |  |  |  |  |  | x |
|  | *Graphiurus kelleni* |  |  |  |  | x |  |  |  |  |  | x |  |
|  | *Graphiurus lorraineus* |  |  |  |  |  |  |  |  |  |  | x |  |
|  | *Graphiurus murinus* | x |  | x | x | x | x | x | x | x | x | x | x |
|  | *Heliophobius argenteocinereus* |  |  | x |  | x | x |  |  |  |  | x |  |
|  |  | KK | | MM | | NR | | NV | | SB | | TV | |
| Size | Scientific Name | H | M | H | M | H | M | H | M | H | M | H | M |
| Sm | *Heliosciurus rufobrachium* | x | x |  | x |  |  |  |  |  |  |  |  |
|  | *Helogale hirtula* |  |  |  | x |  | x |  |  | x | x | x | x |
|  | *Heterocephalus glaber* |  |  | x |  |  |  |  |  | x | x |  | x |
|  | *Heterohyrax brucei* |  |  | x | x | x | x |  | x |  | x | x | x |
|  | *Hylomyscus denniae* |  |  |  |  |  |  | x |  |  |  |  |  |
|  | *Hylomyscus stella* | x | x |  |  |  |  |  |  |  |  |  |  |
|  | *Ichneumia albicauda* | x | x |  | x | x | x | x | x |  | x | x | x |
|  | *Ictonyx striatus* |  |  |  | x | x | x | x |  |  | x |  | x |
|  | *Lemniscomys barbarus* |  |  |  |  | x |  |  |  |  |  | x |  |
|  | *Lemniscomys griselda* |  |  |  |  |  |  |  |  |  |  | x |  |
|  | *Lemniscomys rosalia* |  |  |  |  |  |  |  |  |  |  | x |  |
|  | *Lemniscomys striatus* | x | x | x | x | x | x | x | x | x | x |  |  |
|  | *Lemniscomys zebra* |  |  |  |  | x |  |  |  |  |  |  |  |
|  | *Lepus capensis* |  |  |  | x | x | x | x | x | x | x |  | x |
|  | *Lepus microtis* |  |  | x | x | x |  |  |  | x | x | x |  |
|  | *Lophiomys imhausii* |  |  |  |  |  |  | x |  |  |  |  |  |
|  | *Lophuromys aquilus* | x | x | x |  |  | x | x |  | x | x |  |  |
|  | *Lophuromys flavopunctatus* | x | x | x |  |  | x | x |  |  |  |  |  |
|  | *Lophuromys sikapusi* | x | x |  |  |  |  |  | x |  |  |  |  |
|  | *Manis tricuspis* |  | x |  |  |  |  |  |  |  |  |  |  |
|  | *Mastomys natalensis* | x | x |  |  | x | x | x | x |  | x | x | x |
|  | *Mastomys pernanus* |  |  |  |  |  |  |  |  |  |  |  | x |
|  | *Mastomys spp.* | x |  |  | x |  |  |  |  |  |  |  |  |
|  | *Mungos mungo* |  | x | x | x |  |  |  | x |  | x |  | x |
|  | *Mus bufo* |  | x |  |  |  |  |  |  |  |  |  |  |
|  | *Mus mahomet* |  |  |  |  |  |  |  | x |  |  |  |  |
|  | *Mus minutoides* |  |  |  | x | x |  | x | x |  |  |  | x |
|  | *Mus musculoides* | x | x |  | x | x | x | x | x |  |  | x |  |
|  | *Mus setulosus* |  |  |  | x |  |  |  |  |  |  |  |  |
|  | *Mus spp.* |  | x |  |  | x | x | x | x |  |  |  |  |
|  | *Mus triton* | x |  |  |  | x |  | x | x | x | x |  | x |
|  | *Mylomys dybowskii* | x |  |  |  |  |  |  |  |  |  |  |  |
|  | *Myomyscus brockmani* |  |  | x |  | x |  | x |  |  |  | x |  |
|  | *Nandinia binotata* | x | x |  |  | x | x |  |  |  |  |  | x |
|  | *Oenomys hypoxanthus* | x | x |  |  | x | x | x |  |  |  |  |  |
|  | *Otocyon megalotis* |  |  | x | x |  | x | x | x | x | x |  | x |
|  | *Otolemur crassicaudatus* |  | x |  | x |  | x |  |  |  | x |  | x |
|  | *Otolemur garnettii* |  |  | x |  |  |  |  |  |  |  | x | x |
|  | *Otomys angoniensis* |  |  | x |  | x | x | x |  |  |  |  |  |
|  | *Otomys irroratus* |  | x |  |  |  |  |  |  |  |  |  |  |
|  | *Otomys spp.* |  |  |  |  |  |  |  |  |  | x |  |  |
|  | *Otomys tropicalis* | x |  |  |  |  | x | x |  |  |  |  |  |
|  | *Otomys typus* |  | x |  |  |  |  |  |  |  |  |  |  |
|  | *Paraxerus ochraceus* |  |  |  | x | x | x | x | x | x | x | x | x |
|  | *Pedetes surdaster* |  |  | x | x | x | x | x | x |  | x |  | x |
|  | *Pelomys fallax* |  |  |  | x |  | x |  | x |  |  | x | x |
|  | *Pelomys minor* |  |  |  | x |  |  |  |  |  |  |  |  |
|  | *Perodicticus potto* | x | x |  |  |  |  |  |  |  |  |  |  |
|  | *Petrodromus tetradactylus* |  |  |  |  |  |  |  |  |  |  |  | x |
|  | *Potamogale velox* |  | x |  |  |  |  |  |  |  |  |  |  |
|  | *Praomys delectorum* |  | x |  |  |  |  |  |  |  |  | x | x |
|  | *Praomys jacksoni* | x | x | x | x |  | x | x |  |  |  |  |  |
|  | *Praomys misonnei* |  | x |  |  |  |  |  |  |  |  |  |  |
|  | *Praomys spp.* |  | x |  |  |  |  |  |  |  |  |  |  |
|  | *Praomys tullbergi* |  | x |  |  |  |  | x |  |  |  |  |  |
|  |  | KK | | MM | | NR | | NV | | SB | | TV | |
| Size | Scientific Name | H | M | H | M | H | M | H | M | H | M | H | M |
| Sm | *Procavia capensis* |  |  |  | x |  | x | x | x | x | x |  | x |
|  | *Protoxerus stangeri* | x | x |  | x |  |  |  |  |  | x |  |  |
|  | *Rattus rattus* |  |  | x | x | x |  |  | x |  |  |  |  |
|  | *Rhabdomys dilectus* |  |  |  |  |  |  | x |  |  |  |  |  |
|  | *Rhabdomys pumilio* |  |  |  |  |  |  | x | x | x |  |  |  |
|  | *Saccostomus mearnsi* |  |  | x | x |  |  |  |  | x |  |  |  |
|  | *Sciurus vulgaris* |  |  |  |  |  |  |  |  |  | x |  | x |
|  | *Steatomys parvus* |  |  |  |  | x |  |  |  |  |  |  |  |
|  | *Steatomys spp.* |  |  |  | x |  |  |  |  |  |  |  |  |
|  | *Suncus aequatorius* |  |  |  |  |  |  |  |  |  |  |  | x |
|  | *Suncus megalura* | x | x |  |  |  |  |  |  |  |  |  | x |
|  | *Suncus spp.* |  |  |  | x |  |  |  |  |  |  |  |  |
|  | *Sylvisorex granti* |  |  |  |  |  |  | x |  |  |  |  |  |
|  | *Tachyorychtes spp.* |  |  |  |  |  |  |  |  | x |  |  |  |
|  | *Tachyoryctes annectens* |  |  |  |  |  |  |  | x |  |  |  |  |
|  | *Tachyoryctes ibeanus* |  |  |  | x |  | x |  |  |  |  |  | x |
|  | *Tachyoryctes naivashae* |  |  | x |  |  |  | x | x | x |  |  |  |
|  | *Tachyoryctes ruddi* | x |  |  |  |  |  |  |  |  |  |  |  |
|  | *Tachyoryctes spalacinus* |  |  |  |  |  |  |  |  |  | x |  |  |
|  | *Tachyoryctes splendens* |  |  |  |  | x | x | x | x |  |  |  |  |
|  | *Taterillus emini* |  |  |  |  |  |  |  |  |  |  |  | x |
|  | *Taterillus harringtoni* |  |  |  |  |  |  |  |  |  | x |  | x |
|  | *Thallomys loringi* |  |  | x |  |  |  | x |  |  |  |  |  |
|  | *Thallomys paedulcus* |  |  |  | x |  |  |  |  |  |  |  |  |
|  | *Thryonomys gregorianus* | x |  |  |  |  | x |  |  |  |  | x |  |
|  | *Thryonomys swinderianus* |  |  |  | x |  | x |  |  |  |  |  | x |
|  | *Xerus erythropus* | x |  | x | x |  | x |  | x |  | x | x | x |
|  | *Xerus rutilus* |  |  |  | x |  |  |  |  | x | x |  | x |
|  | *Zelotomys hildegardeae* |  |  |  | x | x |  |  |  |  |  | x |  |
| Sm Total | | 52 | 55 | 35 | 52 | 55 | 56 | 54 | 35 | 36 | 43 | 52 | 68 |

**Table S5.** **Full species lists for each park** (N=244 total, 192 historical and 208 modern). Key: KK = Kakamega, MM = Maasai Mara, NR = Nairobi, NV = Naivasha, SB = Samburu, TV = Tsavo, M = Modern, H = Historic, Lg = Large mammals, Md = Medium-sized mammals, Sm = Small mammals. An ‘x’ indicates confirmed species (see Appendix A for details). Full dataset including all categorizations and unconfirmed data is available from the authors and submitted as a data paper to Ecological Archives [2].

| **Locality** | **Body Size Comparisons** | | **Trophic Comparisons** | |
| --- | --- | --- | --- | --- |
|  | D | p | D | p |
| Kakamega | 12.713 | **0.004** | 2.25 | 0.649 |
| Masaai Mara | 1.494 | 0.948 | 4.00 | 0.271 |
| Nairobi | 1.715 | 0.848 | 2.25 | 0.649 |
| Samburu | 4.451 | 0.216 | 1.00 | >0.999 |
| Tsavo | 2.691 | 0.521 | 2.25 | 0.649 |
| Naivasha | 10.134 | **0.013** | 2.25 | 0.649 |

**TableS6. Significance Test Results for Body Size and Trophic Distributions.**

|  | **ALL** | | | | **SMALL** | | | | | |
| --- | --- | --- | --- | --- | --- | --- | --- | --- | --- | --- |
|  | y | R2 | F | p | y | R2 | F | | p | |
| **All** | y = .177 - .057X | 0.096 | 12.65 | 0.0005 | y = .401 - .141X | 0.209 | 16.683 | | 0.0001 | |
| **Static & Incr** | y = .211 - .007X | 0.003 | 0.256 | 0.614 | y = .365 - .062X | 0.091 | 4.081 | | 0.05 | |
| **Increasing** | y = .271 + .005X | 0.002 | 0.125 | 0.724 | y = .343 - .021X | 0.011 | 0.359 | | 0.553 | |
|  | **MEDIUM** | | | | **LARGE** | | | | | |
|  | y | R2 | F | p | y | R2 | | F | | p |
| **All** | y = .271 - .055X | 0.205 | 2.314 | 0.163 | y = .117 - .017 X | 0.005 | | 0.196 | | 0.661 |
| **Static & Incr** | y = .271 - .055X | 0.205 | 2.314 | 0.163 | y = .167 - .036X | 0.028 | | 1.053 | | 0.311 |
| **Increasing** | y = .282 - .042X | 0.211 | 2.144 | 0.181 | y = .253 + .004X | 4.93E-4 | | 0.011 | | 0.916 |

**Table S7. Regressions for Occupancy vs. Population Density Analysis.**

References:

1. NMNH Collections, Smithsonian Institution. Available: <http://collections.nmnh.si.edu/search/mammals/>. Accessed 21 November 2013.
2. Toth AB, Lyons SK, Behrensmeyer AK (2013) Mammals of Kenya’s Protected Areas. Ecological Archives (*in review*).
