# Supplementary material for: A Century of Change in Kenya's Mammal Communities: Increased Richness and Decreased Uniqueness in Six Protected Areas
Source: PLoS One. 2014 Apr 9;9(4):e93092. doi: 10.1371/journal.pone.0093092 (PMC3981716; doi:10.1371/journal.pone.0093092)
Supplement: File S2 — Appendices A–C: Metadata, Taxonomic Notes, and Site Descriptions. (DOCX) [file pone.0093092.s010.docx]

**Text Appendices**

**Appendix A: Details of methods for assembling species lists by site (updated version of this metadata submitted to Ecological Archives as a Datapaper)**

All historical data were obtained from museum records, including specimen data and written records of sightings [13-14, 26-30]. Most historical data came from the National Museum of Natural History (NMNH) collections of the Smithsonian African Expedition [14], but other museum collections were also searched through the Mammal Networked Information System [13]. To improve data completeness, we included data from the immediate vicinity of each current park (i.e. Hell’s Gate data included in Naivasha, Nandi Forest included in Kakamega, Buffalo springs area included in Samburu). These also were included in area estimates. For historical data, it is often difficult to accurately estimate the exact location of a site, but we were consistent about what historic locality names to include in what parks (e.g., we always placed specimens from “North Ewaso Ngiro River” into the database for Samburu Game Reserve). We also recorded data from further afield (no more than 20 km) for each site, but marked these as “vicinity” specimens and used them in the analysis. This designation indicates that we are lacking a specimen or photo of the species at the site, but expect that they were found in the park, taking into account the preferred habitat and range size for the species. These steps were taken for modern data as well. Vicinity specimens were included in the analysis. The cutoff between historic and modern occurrence was 1950.

Modern records were collected from a combination of museum specimens, park websites [31] species list projects [15-18, 24], Journal articles [1-8, 10, 48, 56-65], books [19-23, 47, 49, 66-68], Wikipedia [69-75], and the IUCN red list [32]. Specimen data was considered conclusive if there were no major taxonomic issues (e.g., a subspecies raised to species level). We avoided placing any species on the list without specimen confirmation or the consensus of several sources. Species lists from the Kenya Wildlife Service [31] and Wikipedia [69-75] generally included only charismatic megafauna and were easily verifiable with specimens, geo-referenced photographs [76], or multiple written accounts. Species lists from African Protected Areas Assessment Tool (APAAT) [18] were based upon range maps from the African Mammals Databank [17] and the IUCN red list [32]. While detailed range maps can be useful in identifying isolated populations, they often generalize ranges. Therefore, all species lists in APAAT were verified by searching for geo-referenced photographs, range descriptions, and books [19-23*,* 47, 49, 66-68]. For example, the widespread range of the Golden jackal, *Canis aureus*, includes Kakamega forest [32], but this species generally inhabits savanna and shrubland, not dense tropical forest. Though Kingdon (1977) excludes the forest proper from its range, several papers cite this species in the environs of the forest, and thus *C. aureus* was included on the “vicinity” list for Kakamega. We took similar steps for several mountainous species (i.e. Klipspringer, Jackson’s Mongoose), which occur in the escarpments between Mt Elgon and Mt. Kenya, but not proven in Kakamega forest [32*,* 22-23, 49]. These species were included as “vicinity” species on the Kakamega list. The IUCN red list indicated an isolated pocket of *Cephalophus weynsi* (Weyns’s Duiker) specifically in Kakamega, with citation, and therefore this species was included. Without such specific evidence, we avoided including species on any list based solely on a generalized range map (e.g., *Canis aureus*). We searched the web to find evidence of modern data for any historical species lacking it. For example, because Lake Naivasha is traditionally a collection site for birds or micro-mammals, museum records to confirm the presence of certain primates do not exist. However, visiting tourists often take photos of a primate if they see one and later georeference the pictures online. Therefore, the presence of the primate can be verified in the modern time period. In order to be used as an occurrence, a photo record had to be easily identifiable as the mammal it claimed to depict, be georeferenced or explicitly stated to originate from one of our sites, and have no indications that the site was labeled incorrectly (i.e. too arid, etc.). Kingdon’s data were included when explicit sighting was shown, or when the range maps were very detailed (e.g. including montane patches around rift valley but excluding lowlands). Data from museum catalogs was used unless taxonomy was unclear. Occurrences listed in published literature were also accepted as true, especially if the study included a survey. The location of a survey had to be specifically stated and had to match with one of our study areas. Occurrences listed from secondhand sources were examined before inclusion. On occasion, we could not find proof of occupancy for relatively widespread mammals that we expected to find at specific sites based on their ranges and habitat requirements. In some cases the species was more elusive, and in other cases they were simply very mobile. Some examples include *Caracal caracal* in Samburu Reserve, and *Hystrix africaeaustralis* near Lake Naivasha. Confirmed *Mellivora capensis* and *Manis temmincki* records were missing from both time periods at various sites, likely because these animals are unusual sightings despite their alleged commonness. Because these missing records could not be proven, we did not include them in the analysis. These unconfirmed records comprise 6% of the historical species lists and 3% of the modern lists and are thus unlikely to impact the results. Of the 208 species in the modern lists, 17 species have unconfirmed records for at least 1 site. However, no species has an unconfirmed record at more than two sites. Of the 238 species in the historical lists, 29 have unconfirmed records for at least 1 site. Only *H. africaeaustralis* has unconfirmed records for more than 2 sites. It is recorded as unconfirmed at all 6 sites in the historical time period, but only because of its widespread range and generalist habitat requirements in the present. We have no proof for its presence in any historical site and therefore it was excluded from historical sites to be conservative. It should be noted that our samples are time averaged samples spanning two 50 year periods. Despite extensive scouring of the literature and web sources, we were unable to find a single confirmed record of an individual of any of these species at the parks in question. Unconfirmed species records were not included in the analyses.

Taxonomy was standardized to Wilson and Reeder (2005) [25], supplemented by additional specific divisions in a few cases, e.g., where IUCN recognized distinct allopatric ranges (e.g. for *Cephalophus harveyi*). This process sometimes required that species be searched under their old name in databases. In both modern and historic data we recognized new divisions (i.e. a subspecies being changed to another species, or raised to a species) but made note if the species (previously a subspecies) was described as differing in certain ecomorphic characteristics from its old group, if no species-specific data was available for the new species. Most historic collectors recorded subspecies names when the taxonomy of the specimen was not certain, making tracing such species through taxonomic changes fairly simple if the subspecies name was retained or recorded in synonymy. Subspecies were otherwise ignored.

Taxonomy issues were addressed to the best of our abilities. In some cases, the only hint of incorrect identification was the general range maps of two closely related species, which do not often overlap. For many of these cases, both species would have been recorded in Kenya, but likely only one actually occurred because its modern range was somewhere else (e.g., *Chlorocebus aethiops* v. *C. pygerythrus*; *Pedetes capensis* v. *P. surdaster*). When this happened, we limited our list to only one of the two species for any park that was not directly on the boundary of the two species ranges. For example, we took all Kenyan records of *Pedetes capensis* to be *P. surdaster*, justifying this because *P. capensis* currently occurs only in Southern Africa, and furthermore, *P. surdaster* was considered a subspecies of *P. capensis* in some historic records. In other cases, a single species showed a specimen distant from its range, and for these we marked the record as a possible mis-identification or anomaly (see Appendix B).

When a genus but not species identification was known for a specimen or record, we recorded the genus on the list, unless a species of that genus was already on that list for the other time period, in which case we recorded the presence of the genus under that species. While we have no way of knowing this is actually true, it is conservative with respect to the null hypothesis that no change in species richness has occurred.

**Appendix B: Taxonomic Notes on species lists**

*Cephalophus*: Records include *adersi, callipygus, harveyi*, *natalensis*, *nigrifrons*, *silvicultor, weynsi*.

--Kingdon and some historic records lump *C. weynsi* under *C. callipygus*, though Wilson and Reeder [25] and IUCN [32] separate *C. weynsi* as a species, and divide the range of the two at the Ubangi/Congo Rivers (the border of Congo and D.R. of the Congo).

--Wilson and Reeder [25] and some historic records lump *C. harveyi* under *C. natalensis*. Both Kingdon [22] and IUCN recognize both species. Border of their ranges is in southern Tanzania.

****Action taken**: Follow IUCN on both counts, recognizing all species divisions.

(1)All *C. callipygus* records in Kenya modified to *C. weynsi*

(2) All *C. natalensis* records in Kenya modified to *C. harveyi*

(3) Master list for Kenya contains *adersi, harveyi, nigrifrons, silvicultor, weynsi.*

*Cercopithecus:* Kenya records include *albogularis, ascanius, mitis, neglectus, nictitans,aethiops, pygerythrus, sabaeus, tantalus.*

--All modern sources agree that *aethiops, pygerythrus, sabaeus, and tantalus* are *Chlorocebus* (see below).

--IUCN lists *nictitans* as western species only. Kingdon lists *nictitans* as superspecies of *nictitans, mitis* and *albogularis*, with *nictitans* as western species only.

--Kingdon and IUCN recognize *albogularis* as a subspecies of *mitis.* Wilson and Reeder separate *albogularis.*

--Kingdon and IUCN recognize *ascanius* and *neglectus* as distinct species present in Kakamega.

--Kingdon, IUCN recognize *mitis* as the common Kenya species

****Action taken:**

(1)All *aethiops, pygerythrus, sabaeus,* and *tantalus* records modified to *Chlorocebus.*

(2)All *nictitans* and *albogularis* records treated as *C. mitis.*

(3)Records of *C. neglectus* and *C. ascanius* in Kakamega were retained, but modified to *C. mitis* in sites obviously outside their rainforest habitats.

*Chlorocebus*: Kenya records include *aethiops, pygerythrus, sabaeus, tantalus.*

*--*Kingdon recognizes all as species of “superspecies” *C. aethiops.* His range maps have *pygerythrus* in Kenya, with a small overlap of *tantalus* in the Kakamega area, but no *sabaeus or aethiops.*

--IUCN and Wilson and Reeder recognize all species; ranges match with Kingdon.

****Action taken:**

(1)All *C. sabaeus* treated as mis-identification, (*pygerythrus)*

(2)All *C. aethiops* records in Kenya modified to *pygerythrus*

(3)*C. tantalus* has no specimens, listed unconfirmed in Kakamega.

*Colobus:* Kenya records include *abyssinicus, guereza, polykomos, angolensis*

--Most sources separate species

--Kingdon lists *abyssinicus* as synonym for *guereza.* IUCN does not recognize *abyssinicus.*

--Kingdon and IUCN agree that *polykomos* is solely a western species.

--Kingdon and IUCN agree that *angolensis* is a coastal species, only in Shimba Hills in Kenya.

****Action taken:**

(1)All *C. abyssinicus* and *polykomos* modified to *C. guereza*

(2)All *C. angolensis* records for the present sites modified to *C. guereza.*

*Damaliscus*: Kenya records include *lunatus, korrigum,* and *hunteri*

--Most sources now agree that *hunteri* represents another genus, *Beautragus*.

--IUCN and Kingdon [22, 32] recognize only *D. lunatus*, *korrigum* being a subspecies.

--Wilson and Reeder recognize four species, including *lunatus* and *korrigum.* The former represents the Southern African population (tsessebe), and the latter represents Eastern Africa (topi).

*****Action taken:**

(1) Records of *D. hunteri* or Hunter’s Hartebeest were modified to *B. hunteri*.

(2) *D*. *lunatus* and *korrigum* were recognized as separate species; all *Damaliscus* records *were* modified to *korrigum*. Data users should decide what species divisions to recognize. If this particular division is not recognized by a data user, then occurrences of *korrigum* should be treated as *D. lunatus*.

*Mastomys coucha, Gazella dorcas:* do not range in Kenya, and are unlikely ever to have done so [32].

****Action taken:*** considered error/anomaly. Removed.

*Papio: hamadryas, cyanocephalus, anubis.*

--Most sources do not recognize *hamadryas* in Kenya except in the hottest desert regions, where we had no sites.

***Action taken:** *P. hamadryas* records modified to *P. anubis*.

*Pedetes*: Most records indicate *P. capensis* in Kenya. This is from before the separation of the two subpopulations into different species. East African species became *P. surdaster*, which is “A distinct species … The separation of *surdaster* from *capensis* was …based upon genetic, morphological, and ethological differences between the East African and South African Springhares.” [25]

****Action taken**: All records of *P. capensis* modified to *P. surdaster*.

*Phacochoerus*: Kenya records include *africanus* and *aethiopicus.*

--All sources recognize this species division. The Tana River is cited as the boundary between their ranges in Kenya.

***Action taken:**

(1) Records of *aethiopicus* south of the Tana were modified to *africanus* and vice versa north of the Tana River.

(2) Both records, if they exist, retained in areas of possible overlap (Samburu).

*Potamochoerus*: *porcus* and *larvatus.*

--Most sources recognize species division, and most agree that only *larvatus* should be found in Kenya.

--Kenya records are a mix of both, probably due to mis-identifications or recent separation.

***Action taken:** All *Potamochoerus* records modified to *larvatus* in Kenya.

**Updated genus and/or species names:**

*Felis caracal* >> *Caracal caracal*

*Felis lybica >> Felis silvestris*

*Serval serval* >> *Leptailurus serval*

*Tatera* >> *Gerbilliscus* (in Kenya)

*Herpestes >> Herpestes, Galerella, Atilax*

*Rhynchotragus >> Madoqua*

*Gazella >> Nanger granti, Eudorcas thomsonii (G. gazella* changed to *N. granti*, clerical error)

*Cercopithecus >> Cercopithecus, Chlorocebus*

*Galago >> Galago, Otolemur*

*Strepsiceros, Tragelaphus >> Tragelaphus*

*Felis lybica >> Felis silvestris*

*Nasilio, Elephantulus >> Elephantulus*

*Erinaceus >> Atelerix*

*Rattus, Epimys >> Arvicanthis, Aethomys, Mastomys, Praomys, Hylomyscus, Myomyscus, Rattus*

*Helogale undulata >> Helogale parvla*

**Appendix C: Spatial and Ecological Matching of Modern and Historical Sites**

*1.1: Historical Site Descriptions*

From T.E. Roosevelt’s *African Game Trails* (1910) and Edmund Heller’s original field notes (1909-1911) [26, 28].

**Kakamega, Forest Station, Kaimosi, now Kakamega Forest National Park**

From Heller’s field notes:

“Kaimosi is a mission station without any industrial or native market. It is well situated on a forested hill in the edge of the Kakumega forest. Forest chiefly tropical Uganda trees. Many figs and Spathodea trees seen. Edge of forest flat-topped Acacias. Brush large purple compositae of tree size and Trunfeta bushes. Brakes cover open hills and forest edge.”

**South Ewaso Ng’iro, now Masaai Mara National Reserve**

From *African Game Trails*:

Alluvial plain

“After a day's rest, we pushed on, in two days' easy travelling,

to the Guaso Nyero of the south. Our camps were pleasant, by running streams of swift water; one was really beautiful, in a grassy bend of a rapid little river, by huge African yew-trees, with wooded cliffs in front. It was cool, rainy weather, with overcast skies and misty mornings, so that it seemed strangely unlike the tropics.”

“…wide plain, on which we could see the game grazing as we walked around camp. The alluvial flats bordering the river, and some of the higher plains, were covered with an open forest growth, the most common tree looking exactly like a giant sage-brush, thirty feet high; and there were tall aloes and cactus and flat-topped mimosa.”

“Slowly we walked across the stretches of brown grassland, sometimes treeless, sometimes scantily covered with an open growth of thorn-trees, each branch armed with long spikes, needle-sharp; and among the thorns here and there stood the huge cactus-like euphorbias, shaped like candelabra, groups of tall aloes, and gnarled wild olives of great age, with hoary trunks and twisted branches. Now and then there would be a dry watercourse, with flat-topped acacias bordering it, and perhaps one pool of thick greenish water.” P.164

“We were in the middle of a vast plain, covered with sunscorched grass and here and there a stunted thorn; in the background were isolated barren hills, and the mirage wavered in the distance.” P165

“following a donga, or small watercourse, fringed with big acacias.” p166

“Our next camp was in the middle of the vast plains, by some limestone springs, at one end of a line of dark acacias. There were rocky koppies two or three miles off on either hand. From the tents, and white-topped wagons, we could see the game grazing on the open flats, or among the scattered wizened thorns. The skies were overcast, and the nights cool”

“Our next camp was pitched on a stony plain, by a winding stream-bed still containing an occasional rush-fringed pool of muddy water, fouled by the herds and flocks of the numerous Masai.”

“The night had been cool, but the day was sunny and hot. At first we rode through a broad valley, bounded by high, scrub-covered hills. The banks of the dry stream were fringed with deep green acacias, and here and there in relief against their dark foliage flamed the orange-red flowers of the tall aloe clumps.”

“The Sotik country through which we had hunted was sorely stricken by drought. The grass was short and withered and most of the waterholes were drying up, while both the game and the flocks and herds of the nomad Masai gathered round the watercourses in which there were still occasional muddy pools, and grazed their neighborhood bare of pasturage.”

photos: pg 173, 175, 181, 191

**Athi Kapiti Plains, and Lukenya Hills, now Nairobi National Park**

From *African Game Trails*:

“Our camp was on a bare, dry plain, covered with brown and withered grass. At most hours of the day we could see round about, perhaps a mile or so distant, or less, the game feeding. South of the track the reserve stretched for a long distance; north it went for but a mile, just enough to prevent thoughtless or cruel people from shooting as they went by in the train. There was very little water; what we drank, by the way, was carefully boiled. The drawback to the camp, and to all this plains region, lay in the ticks, which swarmed, and were a scourge to man and beast…For two or three miles our little horses shuffled steadily northward across the desolate flats of short grass until the ground began to rise here and there into low hills, or koppies, with rock-strewn tops. It should have been the rainy season, the season of "the big rains"; but the rains were late, as the parched desolation of the landscape bore witness; nevertheless there were two or three showers that afternoon.”

“We soon began to see game, but the flatness of the country and the absence of all cover made stalking a matter of difficulty; the only bushes were a few sparsely scattered mimosas; stunted things, two or three feet high, scantily leaved, but abounding in bulbous swellings on the twigs, and in long, sharp spikes of thorns.”

“As my horse shuffled forward, under the bright, hot sunlight, across the endless flats or gently rolling slopes of brown and withered grass, I might have been on the plains anywhere, from Texas to Montana; the hills were like our Western buttes; the half-dry watercourses were fringed with trees…”

“The plains were generally covered only with the thick grass on which the great herds of game fed; here and there small thorn-trees grew upon them, but usually so small and scattered as to give no shelter or cover. By the occasional watercourses the trees grew more thickly, and also on the hills and in the valleys between. Most of the trees were mimosas, or of similar kind, usually thorny; but there were giant cactus-like Euphorbias, shaped like candelabra, and named accordingly; and on the higher hills fig trees, wild olives, and many others whose names I do not know, but some of which were stately and beautiful. Many of the mimosas were in bloom, and covered with sweet smelling yellow blossoms. There were many flowers. On the dry plains there were bushes of the color and size of our own sage-brush, covered with flowers like morning glories. There were also wild sweet-peas, on which the ostriches fed; as they did on another plant with a lilac flower of a faint heliotrope fragrance. Among the hills there were masses of singularly fragrant flowers like pink jessamines, growing on bushes sometimes fifteen feet high or over. There were white flowers that smelt like narcissus, blue flowers, red lilies, orange tiger-lilies, and many others of many kinds and colors, while here and there in the pools of the rare rivers grew the sweet-scented purple lotus-lily.”

“It was a settled country … and for this reason all the more interesting…settlement has already begun…plunging into the absolute wilderness. There was much to remind one of conditions in Montana and Wyoming thirty years ago; the ranches planted down among the hills and on the plains still teeming with game…”

Photos: African Game Trails pg 29

From Heller’s Field notes: From Ulu Station to Kapiti Plains: grass green but short…Soil blackish.

Athi and Kapiti plains beautifully green, the grass about 6 inches deep and pools of water along the line due to last week torrential rains. (December 6th, 1911)

**Lake Naivasha, now Lake Naivasha and Hell’s Gate National Park**

From *African Game Trails*: To Lake Naivasha p. 209.

“On our left the tree-clad mountain side hung above us; ravines, with mimosas clustering in them, sundered the foot-hills, and wound until they joined into what looked like rivers; the thick grass grew waist high. It looked like a well-watered country; but it was of porous, volcanic nature, and the soil was a sieve. After nightfall we came to where we hoped to find water; but there was not a drop in the dried pools; and we had to make a waterless camp.”

“Two or three times we crossed singularly beautiful ravines, the trail winding through narrow clefts that were almost tunnels, and along the brinks of sheer cliffs, while the green mat of trees and vines was spangled with many colored flowers. Then we came to barren ridges and bare, dusty plains;”

“It is a lovely sheet of water, surrounded by hills and mountains, the shores broken by rocky promontories, and Indented by papyrus-fringed bays.”

“We spent one entire day with the row-boat in a series of lagoons near camp, which marked an inlet of the lake. We did not get any hippo, but it was a most interesting day. A broad belt of papyrus fringed the lagoons and jutted out between them. The straight green stalks with their feathery heads rose high and close, forming a mass so dense that it was practically impenetrable save where the huge bulk of the hippos had made tunnels. Indeed, even for the hippos it was not readily penetrable. The green monotony of a papyrus swamp becomes wearisome after a while; yet it is very beautiful, for each reed is tall, slender, graceful, with its pale flowering crown; and they are typical of the tropics, and their mere sight suggests a vertical sun and hot, steaming swamps, where great marsh beasts feed and wallow and bellow, amidst a teeming reptilian life. A fringe of papyrus here and there adds much to the beauty of a lake, and also to the beauty of the river pools, where clumps of them grow under the shade of the vine-tangled tropical trees. The open waters of the lagoons were covered with waterlilies, bearing purple or sometimes pink flowers.”

“In many places the road was beautiful, leading among the huge yellow trunks of giant thorn-trees, the ground rising sheer on our left as we cantered along the edge of the lake.”

Hints surrounded by open grassland.

Photos pg. 212, 224

**North Ewaso Ng’iro, Isiolo, Archer’s Post, now Samburu, Buffalo Springs, and Shaba National Reserves**

From *African Game Trails*:

Note: most of our data are probably not directly from these areas, which were described on the way to the Ewaso Ng’iro:

“The camp was beside a cold, rapid stream, one of the head-waters of the Guaso Nyero. It was heavily fringed with thorn timber. To the east the crags and snowfields of Kenia rose from the slow swell of the mountain's base. It should have been the dry season, but there were continual heavy rains, which often turned into torrential downpours. In the overcast mornings as I rode away from camp, it was as cool as if I were riding through the fall weather at home; at noon, if the sun came out, straight overhead, the heat was blazing; and we generally returned to camp at nightfall, drenched with the cold rain. The first heavy storm, the evening we pitched camp, much excited all my followers. Ali came rushing into the tent to tell me that there was 'a big snake up high.’ This certainly seemed worth investigating, and I followed him outside where everybody was looking at the ‘snake,’ which proved to be a huge, funnel-shaped, whirling cloud, careering across the darkened sky. It was a kind of waterspout or cyclone; fortunately it passed to one side of camp.”

“Next day I hunted along the edges of a big swamp… The country looked like a park, with clumps of thorn-trees scattered over the grassy sward.”

“…we encountered a succession of thunder-storms. The rain came down in a deluge, so that the water stood ankle deep on the flats, the lightning flashed continuously on every side, and the terrific peals of thunder made one continuous roll. At first it maddened my horse; but the uninterrupted blaze and roar, just because uninterrupted, ended by making him feel that there was nothing to be done, and he plodded stolidly forward through the driving storm. In this desolate and lonely land the majesty of the storms impressed on the beholder a sense of awe and solemn exaltation. Tossing their crests, and riven by lightning, they gathered in their wrath from every quarter of the heavens, and darkness was before and under them; then, in the lull of a moment, they might break apart, while the sun turned the rain to silver and the rainbows

were set in the sky; but always they gathered again, menacing and mighty, for the promise of the bow was never kept, and ever the clouds returned after the rain. Once as I rode facing Kenia the clouds tore asunder, to right and left, and the mountain towered between, while across its base was flung a radiant arch. But almost at once the many-colored glory was dimmed; for in splendor and terror the storm strode in front, and shrouded all things from sight in thunder-shattered sheets of rain.”

“Next morning we started toward Meru, heading north-east, toward the foot-hills of Kenia. The vegetation changed its character as we rose. By the stream where we had camped grew the great thorn-trees with yellow-green trunks which we had become accustomed to associate with the presence of herds of game. Out on the dry flats were other thorns, wizened little trees, or mere scrawny bushes, with swellings like bulbs on the branches and twigs, and the long thorns far more conspicuous than the scanty foliage; though what there was of this foliage, now brilliant green, was exquisite in hue and form, the sprays of delicate little leaves being as fine as the daintiest lace. On the foot-hills all these thorn-trees vanished. We did not go as high as the forest belt proper (here narrow, while above it the bamboos covered the mountain side), but tongues of juniper forest stretched down along the valleys which we crossed, and there were large patches of coarse deer fern, while among many unknown flowers we saw blue lupins, ox-eye daisies, and clover. That night we camped so high that it was really cold, and we welcomed the roaring fires of juniper logs.”

“To the north and west we looked far out over the wide, rolling plains to a wilderness of mountain ranges, barren and jagged. All that day and the next we journeyed eastward, almost on the equator. At noon the overhead sun burned with torrid heat; but with the twilight—short compared to the long northern twilights, but not nearly as short as tropical twilights are often depicted—came the cold, and each night the frost was heavy. The country was untenanted by man. In the afternoon of the third day we began to go downhill, and hour by hour the flora changed. At last we came to a broad belt of woodland, where the strange trees of many kinds grew tall and thick. Among them were camphor-trees, and trees with gouty branch tips, bearing leaves like those of the black walnut, and panicles of lilac flowers, changing into brown seed vessels; and other trees, with clusters of purple flowers, and the seeds or nuts enclosed in hard pods or seed vessels like huge sausages. On the other side of the forest we came suddenly out on the cultivated fields of the Wa-Meru, who, like the Kikuyu, till the soil; and among them, farther down, was Meru boma, its neat, picturesque buildings beautifully placed among green groves and irrigated fields, and looking out from its cool elevation over the hot valleys beneath. It is one of the prettiest spots in East Africa.”

The description of our actual site starts here:

“A long day's march brought us down to the hot country. That evening we pitched our tents by a rapid brook, bordered by palms, whose long, stiff fronds rustled ceaselessly in the wind. Monkeys swung in the tree tops…Next day's march brought us to another small tributary of the Guaso Nyero, a little stream twisting rapidly through the plain, between sheer banks. Here and there it was edged with palms and beds of bulrushes. We pitched the tents close to half a dozen flat-topped thorn-trees.”

“The country was a vast plain bounded on almost every hand by chains of far-off mountains. In the south-west, just beyond the equator, the snows of Kenia lifted toward the sky. To the north the barren ranges were grim with the grimness of the desert. The flats were covered with pale, bleached grass which waved all day long in the wind; for though there were sometimes calms, or changes in the wind, on most of the days we were out it never ceased blowing from some point in the south. In places the parched soil was crumbling and rotten; in other places it was thickly strewn with volcanic stones; there were but few tracts over which a horse could gallop at speed, although neither the rocks nor the rotten soil seemed to hamper the movements of the game. Here and there were treeless stretches. Elsewhere there were occasional palms; and trees thirty or forty feet high, seemingly cactus or aloes, which looked even more like candelabra than the euphorbia which is thus named; and a scattered growth of thorn-trees and bushes. The thorntrees were of many kinds. One bore only a few leathery leaves, the place of foliage being taken by the mass of poisonous-looking, fleshy spines which, together with the ends of the branches, were bright green. The camel-thorn was completely armed with little, sharply hooked thorns which tore whatever they touched, whether flesh or clothes. Then there were the mimosas, with long, straight thorn spikes; they are so plentiful in certain places along the Guaso Nyero that almost all the lions have festering sores in their paws because of the spikes…”

“But there were certain trails which did not fade out. These were the ones which led to water. One such we followed. It led across stretches of grassland, through thin bush, thorny and almost leafless, over tracts of rotten soil, cracked and crumbling, and over other tracts where the unshod horses picked their way gingerly among the masses of sharp-edged volcanic stones. Other trails joined in, and it grew more deeply marked. At last it led to a bend in a little river, where flat shelves of limestone bordered a kind of pool in the current where there were beds of green rushes and a fringe of trees and thorn thickets. This was evidently a favorite drinking-place.”

“The camp to which we thus shifted was on the banks of the Guaso Nyero, on the edge of an open glade in a shady grove of giant mimosas. It was a beautiful camp, and In the soft tropic nights I sat outside my tent and watched the full moon rising through and above the tree tops. There was absolutely no dew at night, by the way. The Guaso Nyero runs across and along the equator, through a desert country, eastward into the dismal Lorian swamp, where It disappears, save in very wet seasons, when it continues to the Tana. At our camp it was a broad, rapid, muddy stream Infested with crocodiles. Along Its banks grew groves of Ivory-nut palms, their fronds fan-shaped, their tall trunks forked twenty or thirty feet from the ground, each stem again forking—something like the antlers of a black-tail buck.”

“It must have been a year since any rain had fallen. The surface of the baked soil was bare and cracked, the sparse tussocks of grass were brittle straw, and the trees and bushes were leafless; but instead of leaves they almost all carried thorns, the worst being those of the wait-a-bit, which tore our clothes, hands, and faces.”

“After leaving this camp we journeyed up the Guaso Nyero for several days. The current was rapid and muddy, and there were beds of reeds and of the tall, graceful papyrus. The country round about was a mass of stony, broken hills, and the river wound down among these, occasionally cutting its way through deep gorges, and its course being continually broken by rapids.”

Photos Pg 280, 300

**Taita Hills, Voi, Mitoto-Andei, Galana, now Tsavo East and West National Parks, Taita hills.**

From Heller’s field notes.

Mitoto-Andei: “Country here densely covered by thick bush and gnarled trees. Soil red, country dry, and trees leafless.”

“At Tsavo River saw the first [doum] palms. They are scattered along the river with the green barked Acacias. Small rocky hills rise above the thick brush here.”

“Large hill within half a mile of station. Made of giant blocks…and heavily forested by thorn trees and euphorbias. A small yucca-like palm, first seen near Maungu grows in compress in one plain to 2-3 feet. Many of the large white tubers seen; the produce a vine similar to a sweet potato in foliage. Maungu to Voi: Brush heavy and green. March 23: Mile 250 below Kni, country dry, grass withered but not burnt, aracias green. White blossomed Aracias in flower. Soil bright red.

Three distinct zones on Taita Hills. 1: Base, 1800 to 2500 feet. Mammals: *Acomys, xerus, Paraxerus, Arvicanthus, Elephantulus, Galago galago, Rhynchotragus*. Trees: black-barked Acacias, Green-barked Thorn Trees, scalloped Euphorbias. 2: Intermediate 2500 to 3500 feet. Mammals: *Pelomys, Arvicanthus dorsalis, Graphurus* (small), *E. hindei, Zelotomys, R. hindei, E. voi, E. nivieventris, Crocidura voi, Cricetomys, thryonomys*. Trees: Krythrine figs, palms. 3: Summit-forest. 3500 to 7000 feet. Mammals: *Epimys endorobae, Crocidura, Cricetomys, Graphurous* (large), *Sciurus, Galago cranicaudus, Cercopithecus albogularis, Tragelaphus, Potamochoerus*. Trees: *Podocarpus*, buttressed tree, Magnolia, Albizzia, Pteris. Vegetation of Sagalla: No palms—summit; no pteris fern or blackberry vines; tall grass; dodonea, abundant shrub; lantana, abundant bush; cassia, abundant bush; *Carissa edulis*, a few bushes seen; fig, small fruited, size of cherry.

*1.2 Modern Site Descriptions*

**Kakamega Forest National Park**

“This part of Kenya receives some of the highest rainfall in the country and annually the precipitation is 2,000 milimetres. Most of this rain falls between April and November, with a short dry season from December to March. Rain falls mostly in the afternoon or early evenings and is often accompanied by heavy thunderstorms. The temperatures does not vary greatly throughout the year, with a mean maximum shade figure of 27˚C and a mean minimum of 15˚C. Practically speaking this means that during the daytime it can be quite hot and since there is high humidity in the air.

“Kakamega forest is the only remnant in Kenya of the once great tropical rainforest that stretched across Central Africa, also known as the Guineo-Congolian forest. About 10,000 years ago when the climate became drier the area of the rainforest shrank to what it was about 250 years ago. With time, much of the indigenous forest has been cut down as people needed the land for farming and Kakamega forest has become an exotic isolated relic of this vast African equatorial jungle, making it a unique habitat in Kenya. To the local Luhya people, Kakamega forest has not only been a generous supplier of firewood and building materials for centuries, medicinal plants are being used to heal all kinds of illnesses. Due to commercial exploitation and increased population in the area the future of the forest was uncertain until two nature reserves (Yala and Isecheno) in 1967 were being created and in 1985 two more reserves in the northern part of the forest (Buyangu and Kisere) were being created, somehow reducing the pressure on the forests natural resources.

“Slowly Kakamega forest has become famous around the world for being home to several hundred species of birds, snakes, lizards, insects, monkeys and other mammals. A choice of walking trails, view points and guided tours enable you to experience some of this fascinating, beautiful and unique forest first hand. Especially in the northern part of the forest you will find virgin forest, intersected only by gurgling streams.

“Kakamega forest is situated at an altitude of 1,500 – 1,700m and covers an area of 240 km2 of which some 40 km2 is designated as a protected forest reserve. The forest is situated about 150 km west of Rift Valley, from which it is separated by highlands stretching from the Cherangani hills in the north to the Mau escarpment in the south. The 2,200 metre high Nandi escarpment, a few kilometres to the east of the forest, forms the western limit of these highlands and as a result all rivers rising in this catchment eventually drain into the 68,000 km2 Lake Victoria in the southwest. The underlying rocks of the forest are associated with ancient gneisses of the Kavirondo and Nyanzian systems as well as basalt, phenolites and gold-bearing quartz veins.” [77]

**Masaai Mara National Reserve**

“Habitats in the Masai Mara are varied, including open rolling grassland, riverine forest, *Acacia* woodland, swamps, non-deciduous thickets, boulder-strewn escarpments, and *Acacia*, *Croton* and *Tarchonanthus* scrub. The permanent Mara and Talek rivers, and their tributaries, flow through the reserve and approximately trisect it. There is a pronounced rainfall gradient from the drier east (with c.800 mm rain/year) to the wetter west (with c.1,200 mm/year).” [71]

**Nairobi National Park**

“Savannah ecosystem comprise of different vegetation types. Open grass plains with scattered acacia bush are predominant. The western side has a highland dry forest and a permanent river with a riverine forest.  To the south are the Athi-Kapiti Plains and Kitengela migration corridor which are important wildlife dispersal areas during the rainy season. Man-made dams within the park have added a further habitat, favourable to certain species of birds and other aquatic biome.” [31]

“The park's predominant environment is open grass plain with scattered *Acacia* bushes. The western uplands of the park have highland [dry forest](http://en.wikipedia.org/wiki/Tropical_and_subtropical_dry_broadleaf_forests) with stands of [*Olea africana*](http://en.wikipedia.org/wiki/Olea), *Croton dichogamus*, [*Brachylaena hutchinsii*](http://en.wikipedia.org/wiki/Brachylaena), and [*Calodendrum*](http://en.wikipedia.org/wiki/Calodendrum). The lower slopes of these areas are grassland. [*Themeda*](http://en.wikipedia.org/wiki/Themeda), [Cypress](http://en.wikipedia.org/wiki/Cypress), [*Digitaria*](http://en.wikipedia.org/wiki/Digitaria), and [*Cynodon*](http://en.wikipedia.org/wiki/Cynodon) species are found in these grassland areas. There are also scattered yellow-barked *Acacia xanthophloea*. There is a [riverine forest](http://en.wikipedia.org/w/index.php?title=Riverine_forest&action=edit&redlink=1) along the permanent river in the south of the park. There are areas of broken bush and deep rocky valleys and gorges within the park. The species in the valleys are predominantly *Acacia* and [*Euphorbia candelabrum*](http://en.wikipedia.org/wiki/Euphorbia_candelabrum). Other tree species include [*Apodytes dimidiata*](http://en.wikipedia.org/wiki/Apodytes_dimidiata), [*Canthium schimperiana*](http://en.wikipedia.org/wiki/Canthium), [*Elaeodendron buchananii*](http://en.wikipedia.org/w/index.php?title=Elaeodendron_buchananii&action=edit&redlink=1), [*Ficus eriocarpa*](http://en.wikipedia.org/wiki/Ficus), [*Aspilia mossambicensis*](http://en.wikipedia.org/w/index.php?title=Aspilia_mossambicensis&action=edit&redlink=1), [*Rhus natalensis*](http://en.wikipedia.org/wiki/Sumac), and [*Newtonia*](http://en.wikipedia.org/wiki/Newtonia_(plant)) species. Several plants that grow on the rocky hillsides are unique to the Nairobi area. These species include [*Euphorbia brevitorta*](http://en.wikipedia.org/wiki/Spurge), [*Drimia calcarata*](http://en.wikipedia.org/w/index.php?title=Drimia_calcarata&action=edit&redlink=1), and [*Murdannia clarkeana*](http://en.wikipedia.org/w/index.php?title=Murdannia_clarkeana&action=edit&redlink=1).

The park covers an area of 117.21 square kilometres (28,963 [acres](http://en.wikipedia.org/wiki/Acre)) and is small in comparison to most of [Africa](http://en.wikipedia.org/wiki/Africa)'s national parks. The park's [altitude](http://en.wikipedia.org/wiki/Altitude) ranges between 1,533 metres (5,030 ft) and 1,760 metres (5,774 ft). It has a dry climate. The park is the only protected part of the [Athi-Kapiti](http://en.wikipedia.org/w/index.php?title=Athi-Kapiti&action=edit&redlink=1) [ecosystem](http://en.wikipedia.org/wiki/Ecosystem), making up less than 10% of this ecosystem. The park has a diverse range of habitats and species.

“The park is located about 7 kilometres (4 mi) from the Nairobi's centre. There is electric fencing around the park's northern, eastern, and western boundaries. Its southern boundary is formed by the [Mbagathi River](http://en.wikipedia.org/wiki/Mbagathi_River). This boundary is not fenced and is open to the [Kitengela Conservation Area](http://en.wikipedia.org/w/index.php?title=Kitengela_Conservation_Area&action=edit&redlink=1) (located immediately south of the park) and the Athi-Kapiti plains. There is considerable movement of large [ungulate](http://en.wikipedia.org/wiki/Ungulate) species across this boundary.” [69]

**Lake Naivasha National Park**

“The lake has a surface area of 139 km², and is surrounded by a swamp which covers an area of 64 square km, but this can vary largely depending on rainfall. It is situated at an altitude of 1,884 metres (6,180 ft). The lake has an average depth of 6 m (20 ft), with the deepest area being at Crescent Island, at a maximum depth of 30 m (100 ft). [Njorowa Gorge](http://en.wikipedia.org/w/index.php?title=Njorowa_Gorge&action=edit&redlink=1) used to form the lake's outlet, but it is now high above the lake and forms the entrance to [Hell's Gate National Park](http://en.wikipedia.org/wiki/Hell%27s_Gate_National_Park). [Floriculture](http://en.wikipedia.org/wiki/Floriculture) forms the main industry around the lake. However, the largely unregulated use of lake water for irrigation is reducing the level of the lake and is the subject of concern in Kenya. [Fishing](http://en.wikipedia.org/wiki/Fishing) in the lake is also another source of employment and income for the local population. The lake varies in level greatly and almost dried up entirely in the 1890s. Having refilled, water levels are now dropping again. In 1981, the first geothermal plant for Lake Naivasha was commissioned and by 1985, a total of 45 [MW](http://en.wikipedia.org/wiki/Megawatt) of electricity was being generated in the area. The water level for Lake Naivasha reached a low level of 0.6 m depth in 1945, but the water level rose again, with minor drops, to reach a maximum depth nearly 6 m in 1968. There was another major decline of the water level in 1987, when the depth reached 225 cm above the lake bottom. The decline of the lake water level in 1987 increased concern in the future of [geothermal](http://en.wikipedia.org/wiki/Geothermal_power) industry, and it was speculated that Lake Naivasha underground water might be feeding the geothermal [reservoir](http://en.wikipedia.org/wiki/Reservoir) at Olkaria. Hence, the decline in the lake water would affect the future of the geothermal industry.” [70]

“Lakes are not normally fresh unless water can escape but there is now no visible outlet to Lake Naivasha; the explanation is that there are underground seepages maintaining the movement of fresh water brought into the lake by the Gilgil and Malewa rivers in the north. Only 100 kilometers from [Nairobi](http://www.africanmeccasafaris.com/kenya/guide/nairobi.asp), Lake Naivasha has become a recreational center for Kenyans; there is a flourishing yacht club and many private cabin cruisers and other boats for fishing. All the land surrounding the lake is in private hands so access to the water has to be through the grounds of the hotels on the south-east shore or by the consent of local landowners.” [79]

Area surrounding Lake Naivasha, the source of our data on mammal species, is fairly lush and alternates between agriculture, settlement, open woodland, sparsely wooded savanna, and open grassland. Niches are available for many species including grazers/browsers, woodland species, scavengers, water-dependent small carnivores, etc.

**Samburu National Reserve**

“The reserve lies within ecological zone V – which is classified as arid and semiarid with a moisture index of 42 to 57, which indicates that evapotranspiration is greater than available moisture. The days are extremely hot while the nights are cool.
The annual mean temperatures range between 18º C and 30ºC, while the mean annual rainfall is 345 mm with peaks in November and April.
The dry season starts in late May, and goes up to early October when a large concentration of wildlife is found in the reserve due to availability of lush vegetation along the Ewaso Nyiro River, the main source of water to the Reserve and the nearby communities. In the middle of the reserve, the [Ewaso Ng'iro](http://en.wikipedia.org/wiki/Ewaso_Ng%27iro) flows through [doum palm](http://en.wikipedia.org/wiki/Doum_palm) groves and thick riverine forests that provides water without which the game in the reserve could not survive in the arid country.

Samburu National Reserve is situated on the banks of the Ewaso Ngiro River in the dry northern reaches of Kenya. It is a hot and arid area characterized by a parched landscape of hills and plains.” [79]

**Tsavo National Park**

“Tsavo West National Park is covered in volcanic cones, rocky outcrops and lava flows. The northern part of Tsavo West is the most developed in terms of lodges and infrastructure and has spectacular scenery with a rolling volcanic landscape carpeted in long grass and dense bush.

Tall vegetation makes game spotting here a little trickier than in some of the other parks. The Big Five can be found in the park along with a fine range of antelope species. The main attractions of the park are the two waterholes, built by the lodges to more or less guarantee that their guests will be treated to fabulous game viewing.”

Tsavo East: “The scrub-covered hills of the southern park have a very remote feel and the park, despite its great game, does not attract large numbers of tourists. The best game viewing is along the watercourses and at the Kanderi swamp, which is not far from the main Voi gate. Thirty kilometres from the gate is the Aruba Dam and lion are commonly spotted around here.” [79]

*2.1: Spatial Matching of Historic and Modern Sites.*

Maps of the route taken by the Smithsonian African expedition are available from Heller and Roosevelt’s publication, Life-Histories of African Game Animals (1914), and descriptions are available from Roosevelt’s subsequent book, African Game Trails (1927). The historical equivalents to our modern sites are as follows: specimens taken in the Athi-Kapiti plains are listed under Nairobi National Park. This surveyed area probably comprises the area south of the current park, with some coverage of the current area. We also included specimens from the Lukenya Hills area, to account for the fact that most hunting and trapping in the Athi River area took place only on the plains.

The sampling from Maasai Mara took place through a series of camps chiefly in the Loita Plains, most of which is currently pastoral land from which large non-domestic ungulates are largely excluded. Though they have been pushed out of this historical range, there is evidence that most of these species have been compressed instead into the protected park area, which probably still harbors much of the same fauna in reduced numbers [10], and still contains enough hill habitat to have been a sanctuary even for upland species. The collectors mention that even in the early 1900s, the impact of cattle grazing could be seen in many areas where the grass was cropped completely, and pools of water were muddied and soiled, so this aspect of the sites is consistent across time. It’s important to note that the expedition took place less than 25 years after the start of the rinderpest epidemic in 1889, suggesting that ungulate populations may have been lower than normal at the time of sampling, though lack of actual extinctions means that this probably did not affect presence-absence data.

Lake Naivasha is a significant static landmark, making our assessment of historical sampling in that area fairly dependable. We included data from the vicinity of the lake, which justifies using Hell’s Gate Park as a supplementary data source in the modern record. We deliberately excluded data collected from farther afield, especially those specimens collected in the escarpments of the rift valley near the lake. To maintain consistency, we did not include any modern records of uniquely upland forest species (such as some duikers) in Naivasha records, unless there was some concrete and irrefutable evidence to the contrary, such as a photograph. One population of duikers, *Cephalophus weynsi,* is confirmed by IUCN to have a presence in the low foothills adjacent to the northwestern edge of the lake and cited by Kingdon (1997) as occasionally present in lowlands, so this species we included.

Kakamega sampling was based at Kaimosi mission, which was described as located on “a forested hill in the edge of the Kakumega forest” by E. Heller in his field notes. Today, Kaimosi is a small settlement less than 2 miles from the edge of the protected national forest, but surrounded by small forest fragments that are probably the remnants of the once-continuous habitat. It is not clear from the historical narratives what percent of Kakamega’s area was explored thoroughly, but the area today is so small that we feel it’s likely to be closely analogous to the area historical collectors would have been able to explore on foot.

Modern Samburu was perhaps the largest historical collection zone, the collectors completing a round trip up the Isiolo river to the Northern Ewaso Ng’iro, following it for some distance, then looping back down to Meru and Mt. Kenya. We were careful to include only those data from along the two sampled rivers and avoided the data from farther south. Though the historical collection area was larger than Samburu Reserve, the combination of Samburu, Buffalo Springs, and Shaba reserves in the modern data are probably adequate to make up for this discrepancy.

The first survey of the Tsavo area took place when the collectors rode a train from Mombasa to Nairobi and wrote down what game they saw along the way. In some cases the species name is not mentioned (e.g. “a hyena”), in which case we recorded the species most consistent with the modern record to avoid a bias away from the null hypothesis. The collectors wrote down their observed game over the course of several hours. Later in the expedition, Heller spent several weeks in the Taita Hills outside of Voi, trapping small mammals and recording sightings of large mammals. The Taveta district next to Tsavo West is a popular sampling area, with numerous specimens from both time periods available from a variety of collections. We believe the small mammal collections from this area are very comparable over time.

Because the modern areas tend to be smaller than the historical areas (with the exception of Tsavo), the difference in area is conservative with respect to our results, i.e., smaller areas should have fewer species and increase beta diversity. We found the opposite: increased richness and less beta diversity between parks.

References

1. Ogutu JO, Piepho HP, Dublin HT, Bhola N, Reid RS (2009) Dynamics of Mara–Serengeti ungulates in relation to land use changes. Journ of Zool 278: 1–14.
2. Goldson J (1993) A three phase environmental impact study of recent developments around Lake Naivasha. Lake Naivasha Riparian Owners’ Association, Naivasha 109.
3. Mitchell N (2009) Kakamega Forest Ecosystem: An Introduction to the Natural History and the Human Context. BIOTA East Africa Report. (Univ. of Aööied Sciences, Faculty of Geomatics).
4. Sinclair AR, Mduma SA, Hopcraft JG, Fryxell JM, Hilborn R, et al. (2007) Long-term ecosystem dynamics in the Serengeti: Lessons for conservation. Cons Bio 21: 580–590.
5. Sinclair AR, Packer C, Mduma SAR., Fryxell J. M. (2008) Serengeti III: Human Impacts on Ecosystem. Dynamics. Chicago: University of Chicago Press. 522 p.
6. Western D, Behrensmeyer AK (2009) Bone assemblages track animal community structure over 40 years in an African savanna ecosystem. Science 324: 1061–1064.
7. Kokwaro JO (1988) Conservation status of the Kakamega Forest in Kenya: the easternmost relic of the equatorial rain forests of Africa. Monographs in Systematic Botany of the Missouri Botanical Garden 25: 471–489.
8. Bleher A, Uster D, Bergsdorf T (2006) Assessment of threat status and management effectiveness in Kakamega Forest, Kenya. Biodiv. Cons. 15: 1159–1177.
9. Becht R, Harper DM (2002) Towards an understanding of human impact upon the hydrology of Lake Naivasha, Kenya. Hydrobiol 488: 1–11.
10. Ogutu JO, Owen-Smith N, Piepho HP, Said MY (2011) Continuing wildlife population declines and range contraction in the Mara region of Kenya during 1977–2009. J Zool 285: 99–109.
11. Waithaka J (2012) The Kenya Wildlife Service in the 21st Century: Protecting Globally Significant Areas and Resources 29: 21–29.
12. IUCN, UNEP (2013) The World Database on Protected Areas (WDPA). UNEP-WCMC. Cambridge, UK. Available: [www.protectedplanet.net](http://www.protectedplanet.net). Accessed 10 March 2014.
13. Mammal Networked Information System. Available: <http://manisnet.org/>. Accessed 18 September 2012.
14. NMNH Collections, Smithsonian Institution. Available: <http://collections.nmnh.si.edu/search/mammals/>. Accessed 21 November 2013.
15. Global Biodiversity Information Facility (2013). Available: <http://www.gbif.org>. Accessed 12 January 2013.
16. East R (1999) African antelope database 1998. (World Conservation Union).
17. African Mammals Databank (1999), IEA (Institute of Applied Ecology, Italy**).** Available: [http://www.gisbau.uniroma1.it/amd.php](http://www.gisbau.uniroma1.it/amd.php). Accessed 2 August 2012.
18. The assessment of African protected areas - European Commission. Available: <http://bioval.jrc.ec.europa.eu/APAAT/>. Accessed 13 January 2014.
19. Kingdon J (1971) East African mammals: An Atlas of Evolution in Africa, Vol 1. London: Academic Press. 456 p.
20. Kingdon J (1974) East African mammals: An Atlas of Evolution in Africa, Vol. IIB Hares and Rodents. London: Academic Press. 704 p.
21. Kingdon J. (1974) East African mammals: An Atlas of Evolution in Africa. Vol. IIA. Insectivores and bats. London: Academic Press. 342 p.
22. Kingdon J. (1982) East African Mammals: An Atlas of Evolution in Africa. Vol. IIID, Bovids. London: Academic Press. 358 p.
23. Kingdon, J. (1988) East African Mammals: An Atlas of Evolution in Africa, Vol. 3, Part A: Carnivores. Chicago: University of Chicago Press, edition. 2.
24. The Copenhagen database of African vertebrates. Available: <http://www.zmuc.dk/commonweb/research/biodata.htm> and http://130.225.211.158/subsaharanafrica/subsaharan.htm. Accessed 15 August 2012.
25. Wilson AE, Reeder DAM (2005) Mammal species of the world: a taxonomic and geographic reference. Baltimore: Johns Hopkins University Press, edition 3.
26. Roosevelt T (1927) African Game Trails: An account of the African wanderings of an American hunter-naturalist. New York: C. Scribner’s Sons). 624 p.
27. Roosevelt T, Heller E (1914) Life-histories of African game animals. New York: C. Scribner’s Sons. 954 p.
28. Heller E. (1909-1911) Smithsonian African Expedition Notes of Edmund Heller. National Museum of Natural History Vertebrate Zoology Archives. Available: <http://vertebrates.si.edu/mammals/>. Accessed 2 June 2011.
29. Loring JA (1909-1910) Smithsonian African Expedition Field Catalog. National Museum of Natural History Vertebrate Zoology Archives. Available: <http://vertebrates.si.edu/mammals/>. Accessed 2 June 2011.
30. Mearns EA (1909-1912) Smithsonian African Expedition Field Catalog. National Museum of Natural History Vertebrate Zoology Archives. Available: <http://vertebrates.si.edu/mammals/>. Accessed 2 June 2011.
31. Kenya Wildlife Service, Overview of Parks and Reserves. Available: <http://www.kws.org/parks/parks_reserves/index.html>. Accessed 6 November 2012.
32. IUCN (2010). The IUCN Red List of Threatened Species. Version 2010.3. Available: [http://www.iucnredlist.org](http://www.iucnredlist.org/). Accessed 5 November 2013.
33. ESRI 2011. ArcGIS Desktop: Release 10. Redlands, California: Environmental Systems Research Institute.
34. Goldewijk KK, Beusen A, M de Vos, G van Drecht (2011) The HYDE 3.1 spatially explicit database of human induced land use change over the past 12,000 years. Global Ecology and Biogeography 20(1): 73-86. [DOI: 10.1111/j.1466-8238.2010.00587.x.](http://onlinelibrary.wiley.com/doi/10.1111/j.1466-8238.2010.00587.x/abstract)
35. Goldewijk KK, Beusen A, Janssen P (2010). Long term dynamic modeling of global population and built-up area in a spatially explicit way, HYDE 3 .1. The Holocene 20(4): 565-573. Available: <http://dx.doi.org/10.1177/0959683609356587> and <http://themasites.pbl.nl/tridion/en/themasites/hyde/index.html>. Accessed 19 Feb 2014.
36. Bouwman, AF., Kram T, and Klein Goldewijk K. (2006) Integrated Modelling of Global Environmental Change, An Overview of IMAGE 2.4, Netherlands Environmental Assessment Agency. MNP, Bilthoven, The Netherlands. Available: IMAGE model: <http://themasites.pbl.nl/tridion/en/themasites/image/index.html>. Accessed 19 February 2014.
37. Smith FA, Lyons SK, Ernest SKM, Jones KE, Kaufman DM, et al. (2003) Body mass of late Quaternary mammals. Ecology 84: 3403–3403.
38. Lyons SK, Smith FA (2013) Macroecological patterns of body size in mammals across time and space in Animal Body Size: linking pattern and process across space, time and taxonomic group, In: Smith FA, Lyons SK, Eds. Chicago: University of Chicago Press. pp.114-142.
39. Marquet PA, Cofre H (1999) Large temporal and spatial scales in the structure of mammalian assemblages in South America: a macroecological approach. Oikos 85(2): 299-309.
40. Bakker V J, Kelt DA (2000) Scale-dependent patterns in body size distributions of neotropical mammals. Ecology 81: 3530–3547.
41. World Resources Institute, Kenya GIS data. Available: <http://www.wri.org/publication/content/9291>. Accessed 15 February 2014.
42. Jones KE, Bielby J, Cardillo M, Fritz SA, O’Dell J, et al. (2009) PanTHERIA: a species-level database of life history, ecology, and geography of extant and recently extinct mammals. Ecology 90: 2648–2648.
43. Hanski I (1982) Dynamics of regional distribution: the core and satellite species hypothesis. Oikos 210-221.
44. Magurran AE, Henderson PA (2003) Explaining the excess of rare species in natural species abundance distributions. Nature 422(6933): 714-716.
45. Harper DM, Boar R, Everard M, Hickley P (2003) Lake Naivasha, Kenya. New York: Springer. 205 p.
46. Woodroffe R., Ginsberg J (1998) Edge effects and the extinction of populations inside protected areas. Science 280: 2126-2128.
47. Sinclair AR, Norton-Griffiths M (1995) Serengeti: dynamics of an ecosystem.Chicago: University of Chicago Press) 389 p.
48. Fashing PJ (2002) Population status of black and white colobus monkeys (*Colobus guereza*) in Kakamega Forest, Kenya: are they really on the decline? African Zoology 37: 2.
49. Kingdon J, Pagel M (1997) The Kingdon field guide to African mammals. London: Academic Press. 488 p.
50. Songa JM, Overholt WA, Mueke JM, Okello RO (2002) Farmers’ perceptions of aspects of maize production systems and pests in semi-arid eastern Kenya: factors influencing occurrence and control of stemborers. International Journal of Pest Management 48: 1–11.
51. Hill CM (1997) Crop-raiding by wild vertebrates: The farmer’s perspective in an agricultural community in western Uganda. International Journal of Pest Management 43: 77–84.
52. Wearn OR, Reuman DC, Ewers RM (2012) Extinction debt and windows of conservation opportunity in the Brazilian Amazon. Science 337: 228-232.
53. Rybicki J, Hanski I (2013) Species-area relationships and extinctions caused by habitat loss and fragmentation. Ecology Letters 16: 27-38.
54. Ngigi TG, Tateishi R (2004) Monitoring deforestation in Kenya. International Journal of Environmental Studies 61: 281-291.
55. Gallant AL, Klaver RW, Casper GS, Lannoo MJ (2007) Global rates of habitat loss and implications for amphibian conservation. Copeia 2007: 967-979.
56. East R (1984) Rainfall, soil nutrient status and biomass of large African savanna mammals. Afr. J. Ecol. 22: 245–270.
57. Coe MJ, Cumming DH, Phillipson J (1976) Biomass and production of large African herbivores in relation to rainfall and primary production. Oecologia 22: 341–354.
58. Butynski TM (2000) Independent evaluation of hirola antelope (*Beatragus hunteri*) conservation status and conservation action in Kenya. Unpublished report. Kenya Wildlife Service and Zoo Atlanta.
59. Evans B (2011) Hirola (*Beautragus hunteri*) population status and depredation impacts in Tsavo East National Park, Kenya. Thesis. Imperial College, London.
60. Hofmann RR (1996) Hirola translocation to Tsavo East NP and new scientific information. Gnusletter 15: 2-5.
61. Gentry AW (1964) Skull characters of African gazelles. The Annals & Magazine of Natural History. 7(78): 353-382.
62. Talbot LM, Stewart DRM (1964) First wildlife census of the entire Serengeti-Mara region, East Africa. J. Wildlife Managmt. 28(4): 815-827.
63. Reed DN (2007) Serengeti micromammals and their implications for Olduvai paleoenvironments in Bobe R, Alemseged Z, Behrensmeyer AK, editors. Hominin environments in the east African Pliocene: An assessment of the faunal evidence, eds. New York: Springer. pp. 217-255.
64. Darling F (1960) An ecological reconnaissance of the Mara Plains in Kenya Colony. Wildlife Monographs 5: 3-41.
65. El Kammah KM, Hoogstraal H, Camicas JL (1992) Notes on African Haemaphysalis ticks: XI. *H.* (Rhipistoma) *paraleachi* (Ixodoidea: Ixodidae) distribution and hosts of adults. Int’l Journ Acarol 18(3): 205-212.
66. Nowak RM, Paradiso JL (1992) Walker’s Mammals of the World. Baltimore: Johns Hopkins Univ. Press. 1947 p.
67. Estes R (1991) The Behavior Guide to African Mammals. Berkeley and Los Angeles: Univ. of California Press. 619 p.
68. JG Williams (1967) A field guide to the national parks of East Africa. London: Collins. 336 p.
69. Nairobi National Park (2012) Wikipedia, the free encyclopedia. Available: http://en.wikipedia.org/wiki/Nairobi_National_Park. Accessed 28 January 2013.
70. Lake Naivasha (2012) Wikipedia, the free encyclopedia. Available: <http://en.wikipedia.org/wiki/Lake_Naivasha>. Accessed 28 January 2013.
71. Maasai Mara (2012) Wikipedia, the free encyclopedia. Available: <http://en.wikipedia.org/wiki/Maasai_Mara>. Accessed 28 January 2013.
72. Kakamega Forest (2012) Wikipedia, the free encyclopedia. Available: <http://en.wikipedia.org/wiki/Kakamega_Forest>. Accessed 28 January 2013.
73. Tsavo East National Park (2012) Wikipedia, the free encyclopedia. Available: http://en.wikipedia.org/wiki/Tsavo_East_National_Park. Accessed 28 January 2013.
74. Tsavo West National Park (2012) Wikipedia, the free encyclopedia. Available: http://en.wikipedia.org/wiki/Tsavo_West_National_Park. Accessed 28 January 2013.
75. Samburu National Reserve (2012) Wikipedia, the free encyclopedia. Available: <http://en.wikipedia.org/wiki/Samburu_National_Reserve>. Accessed 28 January 2013.
76. Google Earth (Version 6.1.0.5001) [Software]. Mountain View, CA, Google Inc. (2009) Available: <http://www.google.com/earth/>. Accessed 28 January 2013.
77. Kakamega forest - remnant of the Guineo-Congolian forest covering Africa. Available: http://www.africa-expert.com/about-kenya/national-parks/kakamega-forest/. Accessed 28 January 2013.
78. The African Great Rift Valley - The Maasai Mara - UNESCO World Heritage Centre. Available: http://whc.unesco.org/en/tentativelists/5512/. Accessed 28 January 2013.
79. Description taken directly from safari websites which all use the same text (i.e. <http://www.go2africa.com/kenya/tsavo-national-park>, http://www.magicalkenya.com/index.php?option=com_content&task=view&id=266&Itemid=221, http://www.africanmeccasafaris.com/kenya/guide/lakenaivasha.asp). Accessed 20 February 2013.
